# The first apicoplast tRNA thiouridylase plays a vital role in the growth of *Toxoplasma gondii*

**DOI:** 10.3389/fcimb.2022.947039

**Published:** 2022-08-15

**Authors:** Yimin Yang, Mi Lin, Xueqiu Chen, XianFeng Zhao, Lulu Chen, Mingxiu Zhao, Chaoqun Yao, Kaiyin Sheng, Yi Yang, Guangxu Ma, Aifang Du

**Affiliations:** ^1^ Institute of Preventive Veterinary Medicine and Zhejiang Provincial Key Laboratory of Preventive Veterinary Medicine, College of Animal Sciences, Zhejiang University, Hangzhou, China; ^2^ Animals & Plant Inspection and Quarantine Technology Center of Shenzhen Customs, Shenzhen, China; ^3^ Department of Biomedical Sciences and One Health Center for Zoonoses and Tropical Veterinary Medicine, Ross University School of Veterinary Medicine, Basseterre, Saint Kitts and Nevis

**Keywords:** apicoplast, 2-thiouridylase, TgMnmA, Toxoplasma gondii, s^2^U34 tRNA modification

## Abstract

Toxoplasmosis caused by the protozoan *Toxoplasma gondii* is one of the most common parasitic diseases in humans and almost all warm-blooded animals. Lys, Glu, and Gln-specific tRNAs contain a super-modified 2-thiourea (s^2^U) derivatives at the position 34, which is essential for all living organisms by maintaining the structural stability and aminoacylation of tRNA, and the precision and efficiency of codon recognition during protein translation. However, the enzyme(s) involved in this modification in *T. gondii* remains elusive. In this report, three putative tRNA-specific 2-thiolation enzymes were identified, of which two were involved in the s^2^U34 modification of tRNA^Lys^, tRNA^Glu^, and tRNA^Gln^. One was named *Tg*MnmA, an apicoplast-located tRNA-specific 2-thiolation enzyme in *T. gondii*. Knockout of *TgMnmA* showed that this enzyme is important for the lytic cycle of tachyzoites. Loss of *Tg*MnmA also led to abnormities in apicoplast biogenesis and severely disturbed apicoplast genomic transcription. Notably, mice survived from the infection with 10 *Tg*MnmA-KO RH tachyzoites. These findings provide new insights into s^2^U34 tRNA modification in Apicomplexa, and suggest *Tg*MnmA, the first apicoplast tRNA thiouridylase identified in all apicomplexans, as a potential drug target.

## Introduction

*Toxoplasma gondii* is an obligate intracellular parasitic protozoan that belongs to the phylum Apicomplexan, which includes other important pathogens such as *Plasmodium* spp. and *Cryptosporidium* spp. The life cycle of *T. gondii* is divided into asexual reproduction in the intermediate host and sexual reproduction in the small intestinal epithelial cells of the definitive host which is the domestic cat and other felids (Kochanowsky and Koshy, 2018). The disease it causes, toxoplasmosis is a zoonosis of medical and veterinary importance worldwide (Montoya and Liesenfeld, 2004). Studies have shown that toxoplasmosis is often an asymptomatic infection in people and animals with competent immunity. However, it can have devastating consequences for those with compromised immunity or pregnancy (Esch, 2010). The high incidence of toxoplasmosis threatens the development of agriculture and animal husbandry and brings huge economic losses globally (Montoya and Liesenfeld, 2004). At present, the standard therapy for toxoplasmosis is a combination of sulfadiazine and pyrimethamine, which has disadvantages such as long course of treatment, toxicity and significant failure rate (Dunay et al., 2018). Accordingly, there is an urgent need to identify new drug targets for the treatment of toxoplasmosis.

All biological macromolecules including proteins, RNA, DNA, sugars and lipids are subject to chemical modification upon biochemical synthesis for functional regulation. Of all known RNA species, ribosomal RNA (rRNA) and transfer RNA (tRNA) are the most heavily modified. There are many post-transcriptional chemical modifications in tRNA on the four basic nucleotides (U, C, G and A) and each tRNA contains eight modifications on average (Phizicky and Alfonzo, 2010). So far, more than 100 types of tRNA modifications have been found in all domains of life, among which sulfur modifications are particularly important (Boccaletto et al., 2018). Sulfur Modifications are essential to maintain the structural stability and aminoacylation of tRNAs, and the precision and efficiency of codon recognition (Shigi, 2014). There are a variety of sulfur-modified nucleotides in tRNAs, which are commonly found at seven different positions: 8, 9, 32, 33, 34, 37, and 54, including 2-thiouridine (s^2^U) derivatives, 4-thiouridine (s^4^U), 2-thiocytidine (s^2^C) and 2-methylthioadenosine (ms^2^A) (Shigi, 2018). Compared to other positions of tRNAs, the nucleotides at the wobble position 34 of the anticodon are more prone to modification. The U34 of tRNA^Gln^_UUG_, tRNA^Lys^_UUU_, and tRNA^Glu^_UUC_ is universally 2-thiolated in all three domains of life, being hypermodified to different s^2^U derivatives (xm^5^s^2^U) (Cavuzic and Liu, 2017; Suzuki, 2021). Their biosynthesis involves a “ sulfur trafficking system” initiated by cysteine desulfurase and a “modification enzyme” that directly incorporates sulfur into tRNAs (Wang et al., 2010). In bacteria, the tRNA modifying enzyme participating in s^2^U modification of tRNA^Lys^, tRNA^Glu^ and tRNA^Gln^ is MnmA (Shigi, 2014), while in archaea, it is NcsA (Chavarria et al., 2014). Mtu1 is involved in the 2-thio group in mitochondrial tRNA^Lys^, tRNA^Glu^ and tRNA^Gln^ but not 2-thiolation of cytoplasmic tRNAs (Umeda et al., 2005). In contrast, the tRNA modifying enzyme involved in 2-thiolation of cytoplasmic tRNAs in eukaryotes is Ncs6 (Cavuzic and Liu, 2017; Suzuki, 2021). Nonetheless, no functional studies of tRNA modifying enzyme(s) responsible for 2-thiolation of tRNAs have been conducted to elucidate their roles in any apicomplexans.

In addition to nuclear DNA, apicomplexans have two extrachromosomal genomes, separately residing in the mitochondrion and apicoplast. Mitochondrion in *T. gondii* tachyzoite contains a minimal genome carrying fragmented rRNA genes and three protein-encoding genes. However, it lacks tRNA genes (Namasivayam et al., 2021). The second DNA-containing organelle is the apicoplast (apicomplexan plastid), a vestigial plastid existing in most of the apicomplexan parasites, which no longer undertakes the function of photosynthesis (Lim and McFadden, 2010). The apicoplast evolved from the secondary endosymbiont event with engulfment of a photosynthetic cyanobacterium (Archibald, 2015), and its genome in *T. gondii* contains 4 rRNAs, 33 tRNAs and 28 predicted protein-coding genes (Seeber et al., 2020) (**Supplementary Table 1**). The presence of tRNAs and the capability of self-protein expression indicate that there are nucleus-encoded tRNA modifying enzymes trafficking to the apicoplast, which play an important role for accurate and efficient protein translation. The apicoplast contains three tRNAs with especially important s^2^U34 modification, including ${\text{tRNA}}_{\text{UUG}}^{\text{Gin}}$ , ${\text{tRNA}}_{\text{UUU}}^{\text{Lys}}$ and ${\text{tRNA}}_{\text{UUC}}^{\text{Glu}}$ , so we are keen to explore the functional thiouridylase involved in 2-thiolation of apicoplast tRNAs here. Indeed, without an apicoplast, *T. gondii* tachyzoites died after one complete intracellular developmental cycle (IDC) (Kennedy et al., 2019). The unique characteristics of apicoplast present possibilities for intervention, i.e., the proteins involved in the replication, transcription and translation of the apicoplast genome are expected to provide a variety of new chemotherapeutic targets.

Here we screened *T. gondii* tRNA s^2^U34 thiouridylases in nuclear, mitochondrion and apicoplast genomes, and identified that the TGGT1_309110 gene is apicoplast-located. TGGT1_309110 has high homology with plant *MnmA*, so we named it as *TgMnmA*. To further investigate the role of *TgMnmA* in *T. gondii*, we generated a *TgMnmA*-KO in RHΔ*ku80* strain by CRISPR*/*Cas9. We elucidated the effect of *Tg*MnmA deficiency on the growth of *T. gondii in vitro* and examined the loss of *Tg*MnmA on parasite adhesion, invasion and replication. *TgMnmA*-KO parasites were further assessed for the impact of *TgMnmA*-KO on apicoplast biogenesis and transcription of the apicoplast genome. The survival of mice infected with *TgMnmA*-KO parasites was investigated.

## Materials and methods

### Bioinformatics analysis

Protein sequences of tRNA s^2^U34 thiouridylases including MnmA, MTU1, NCS6 and NCSA in various species were obtained from NCBI (https://www.ncbi.nlm.nih.gov/), and the homologous tRNA thiouridylases in *T. gondii* were screened by BLAST from the ToxoDB (https://toxodb.org/toxo/). HMMER (https://www.ebi.ac.uk/Tools/hmmer/search/phmmer) was used for domain prediction of tRNA thiouridylases. A bootstrap consensus tree of tRNA s^2^U34 thiouridylases sequences was built with MEGA X (http://www.megasoftware.net/) using Maximum Likelihood, tested by Bootstrap method with 1000 replications.

### Parasite and host cell culture

Parental *T. gondii* RHΔ*ku80*Δ*hxgprt* (here referred as RHΔ*ku80*) and subsequent strains were cultivated in confluent monolayers of human foreskin fibroblasts (HFF) in Dulbecco’s modified Eagle medium (DMEM) (Biological Industries, Israel) supplemented with 2.5% fetal bovine serum (Gibco, USA) and 1% penicillin-streptomycin-glutamine (Gibco). All strains and host cell lines were tested negative for *Mycoplasma* sp. with the One-Step Mycoplasma Detection Kit (Yeason, China). The cells were cultured in a humidified incubator with 5% CO_2_ at 37°C. Tachyzoites used in experiments were freshly purified with a syringe filter of 5-μm pore size (Millipore, Germany).

### Parasite transfection

For each transfection, 10-20 million purified tachyzoites in 300 μL cytomix buffer (Shen et al., 2017) were mixed with 7.5 μg purified CRISPR/Cas9 plasmid and 10 μg donor DNA fragment in a 2 mm gap gene pulser^®^ cuvette (Bio-Rad, USA) and electroporated with the Gene Pulser Xcell electroporator (Bio-Rad). After electroporation, the parasites were immediately transferred to confluent HFF host cells. Transfected parasites were grown in HFF cultures for 24 h for recovery prior to drug selection for stable transformants.

### CRISPR/Cas9 gene tagging

For C-terminal 3HA or 6HA tagging, CRISPR/Cas9 plasmids were constructed to create double stand nicks targeting the gene 3′ UTR near the translation stop codon (Shen et al., 2017). The SgRNA sequences were annealed and ligated with the *Bbs* I-digested pSAG1::Cas9-U6::*Bbs* I plasmid, which was modified from pSAG1::CAS9-U6::sgUPRT (Addgene plasmid 54467). A homology-directed repair template was PCR amplified using 59 bp PCR primers that contain 39 bp fragments upstream of the translation stop codon and downstream of the Cas9 break site from the vector pLinker-BirA-3xHA-HXGPRT-loxP (Addgene plasmid 86668) or pLinker-6xHA-HXGPRT-LoxP (Addgene plasmid 86552) that includes the epitope tag and selection cassette. The plasmid was purified by phenol-chloroform extraction, precipitated in ethanol, and electroporated into RHΔ*ku80* parasites, along with the sequence-verified CRISPR/Cas9 plasmid. Transfected parasites were selected with mycophenolic acid (25 μg/mL) and xanthine (50 μg/mL), and then cloned by limiting dilution. PCR, IFA, and Western blot were performed to confirm successful tagging of clones containing 3HA or 6HA. Primers used in this study were provided in **Supplementary Table 2**.

### CRISPR/Cas9 gene deletion and complementation

For gene deletion, a CRISPR/Cas9 plasmid was generated to cut the gene coding region. This plasmid was co-transfected with a *TgMnmA*-specific knockout fragment that contained an EGFP-DHFR selectable marker, flanked by 40 bp specific 5’ and 3’ homology arms. Transfected parasites were selected with pyrimethamine (3 μM). Knockout clones were then isolated by limiting dilution and verified by PCR and qRT-PCR. For gene complementation, a CRISPR/Cas9 plasmid was generated to target the DHFR region. This plasmid was co-transfected with a *TgMnmA*-specific complementing fragment that contained a HXGPRT selectable marker, flanked by 40 bp specific 5’ and 3’ homology arms. Transfected parasites were selected with mycophenolic acid (25 μg/mL) and xanthine (50 μg/mL), and then cloned by limiting dilution. PCR, IFA, and Western blot were performed to confirm successful insertion.

### Immunofluorescence assays and western blot

For IFA, confluent HFF monolayers grown on glass coverslips were infected with *T. gondii* tachyzoites followed by fixation in 4% formaldehyde. They were then permeabilized with 0.25% Triton X-100 in phosphate-buffered saline (PBS), blocked with 1% bovine serum albumin (BSA)/PBS, labeled with primary antibodies, and washed with PBS. Alexa Fluor 488/594/647-conjugated secondary goat antibodies (1:1000) were used. Nuclei were stained with 4’,6-diamidino-2-phenylindole (DAPI). Coverslips were viewed with a fluorescent microscope (Zeiss LSM 880), and processed with ZEN 2.3 software (Zeiss). Rabbit monoclonal anti-HA Tag (C29F4) antibodies (Cell Signaling Technology, USA) were used at a dilution of 1:500. The primary antibodies: mouse anti-*Tg* (1:2000), mouse anti-IMC1 (1:1000), mouse anti-Cpn60 (1:1000), mouse anti-Sortilin (1:1000), mouse anti-Centrin 1 (1:1000), mouse anti-ROP14 (1:1000), mouse anti-GRA16 (1:1000), rabbit anti-IMC1 (1:1000) and rabbit anti-GAP45 (1:1000), were prepared and preserved in the lab.

For Western blot, purified parasites were lysed in protein lysis buffer with protease inhibitor cocktail. Lysates were resolved by SDS-PAGE and transferred onto polyvinylidene fluoride (PVDF) membranes (Millipore, USA), and proteins were detected with the appropriate primary antibody and corresponding secondary antibody conjugated to horseradish peroxidase. Chemiluminescence was induced using the FDbio-Femto ECL Kit (Fdbio Science, China) and imaged on a ChemiDoc system (Bio-Rad, USA). Western blot signals were analyzed with Image Lab 5.2.0 software.

Primary antibodies were diluted as follows: rabbit anti-HA (Cell Signaling Technology, USA) 1:1000 and mouse anti-SAG1 (prepared and preserved in the lab) 1:1000. HRP-conjugated antibodies were diluted as follows: goat anti-mouse (Fdbio Science, China) 1:5,000 and goat anti-rabbit (Fdbio Science, China) 1:5,000.

### Plaque assay

Freshly harvested tachyzoites (n = 200) were added to six-well plates of confluent HFF monolayers and incubated at 37 °C with 5% CO_2_. On day 7, HFF monolayers were fixed in methanol for 10 min, washed in PBS, and stained with 0.1% crystal violet solution for visualization. Plaque numbers were counted for each well. Areas of plaques were measured by a Pixel plugin in the Photoshop C6S software (Adobe, USA).

### Replication assay

Freshly harvested parasites (n = 1×10^5^) were allowed to invade 24-well plates of confluent HFF monolayers grown on glass coverslips for 3 h, then free parasites were washed off. At 24 h PI, infected HFF monolayers were fixed with 4% paraformaldehyde for 15 min and then permeabilized with 0.25% Triton X-100, blocked with 1% BSA. Cells were incubated with rabbit anti-GAP45 in blocking buffer for 1 h. Next, goat anti-rabbit Alexa-Fluor 488 (Thermo Fisher) was applied for 1 h, followed by antifade mountant with 4′,6-diamidino-2-phenylindole (DAPI) to visualize the nucleus. All samples were anonymized prior to parasite counting. In each sample, 10 image fields at 360× amplification (each containing approximately 15 to 20 vacuoles) were analyzed.

### Attachment and invasion assay

Freshly harvested parasites (n = 5×10^6^) were allowed to attach and invade 24-well plates of HFF monolayers grown on glass coverslips for 30 minutes, then washed to remove non-invaded parasites. Infected HFF monolayers were fixed with 4% paraformaldehyde for 15 min and then blocked with PBS supplemented with 1% BSA. Extracellular parasites were first labeled with mouse anti-*Tg* then washed with PBS. After being washed in PBS, monolayers were labeled with goat anti-mouse IgG Alexa Fluor 594.The monolayers were then permeabilized with 0.25% Triton X-100, blocked with 1% BSA followed by labeling all parasites with rabbit anti-GAP45. After being washed with PBS, monolayers were labeled with goat anti-rabbit IgG Alexa Fluor 488. The nuclei were stained with DAPI. At least 10 images under 1,000× amplification for each sample were taken to count the number of parasites and host nuclei for the attachment and invasion assay. The relative efficiency of invasion was calculated as the mean percentage of invaded parasites to the total number of parasites. The data were obtained from three independent experiments, each containing three technical replicates.

### ATc treatment

*T. gondii* tachyzoites were treated with 4 μg/mL anhydrotetracycline (ATc) to eliminate the apicoplast by blocking its protein biosynthesis (Dahl et al., 2006; Wiesner et al., 2008). Freshly harvested parasites were allowed to attach and invade HFF monolayers grown on glass coverslips for 3 h, and then parasites were treated for 21 h with 4 μg/mL of ATc or with EtOH as a control before fixation with 4% paraformaldehyde. In addition, freshly harvested parasites were allowed to grow for 24 h, at which time 4 μg/mL ATc or EtOH was added. After another 24 hours, HFF monolayers were fixed. IFA using anti-HA, anti-IMC1 and anti-Cpn60 or anti-Sortilin antibodies was performed.

### Measurements of mRNA levels

Total RNA was extracted from purified parasites grown in HFF monolayers for 24 h using TRIzol (Invitrogen, USA), and cDNA was generated using ReverTra Ace qPCR RT Kit (Toyobo, Japan). Quantitative PCR was carried out using specific primers with SYBR^®^ Green Realtime PCR Master Mix (Toyobo, Japan) on a T100 Real-Time PCR System (Bio-Rad, USA). Relative levels of transcripts were calculated using the 2^-ΔΔCT^ method using the tubulin-β gene as an internal control. All experiments were performed at least three times, each with three technical replicates.

### Mice survival assay

Mice were purchased from Shanghai SLAC Laboratory Animal Co., Ltd (Shanghai, China). Female 6- to 8-week-old ICR mice were infected intraperitoneally with freshly harvested tachyzoites of indicated *T. gondii* strains. Each group consisted of 6 mice, and each mouse was given *TgMnmA*-KO or RHΔ*ku80* containing 10, 100, 1000 tachyzoites in 100 μL serum-free medium. The survival of mice was monitored at least twice a day. All surviving mice were confirmed seropositive at 3 weeks PI by ELISA using 2 µg/mL RHΔ*ku80*-soluble antigens.

### Quantification and statistical analysis

Data were presented as the mean ± standard deviation (SD). Statistical comparisons were determined using Student’s t tests or two-way ANOVA in the GraphPad Prism 8.0.1 (GraphPad Software, USA). Comparisons were considered statistically significant as follows: *P< 0.05; **P < 0.01; ***P < 0.001, ****P < 0.0001.

## Results

### *T. gondii* nuclear genome encodes three putative tRNA-specific 2-thiolation enzymes

**Figure 1:**
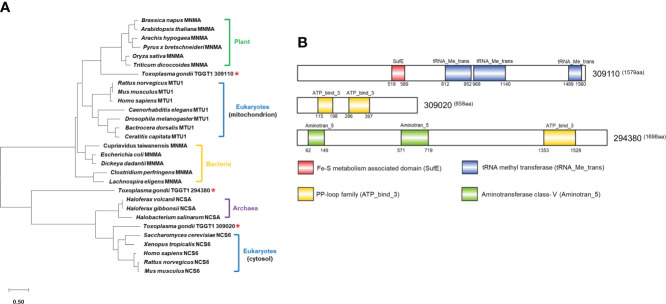
Phylogenetic analysis and domain prediction of putative *Toxoplasma* thiouridylases. **(A)** Phylogenetic tree of tRNA 2-thiolation enzymes in different species. Asterisks indicate homologous proteins in *T. gondii*. The amino acid sequence-based phylogenetic tree was constructed with the Maxium Likelihood algorithm using MEGA 10. Bootstrap analysis was performed with 1,000 replicates. The scale bar indicates the branch length value. Amino acid sequences and accession numbers used to generate phylogenetic tree are presented in **Supplementary File 6**. **(B)** Domain architecture of TGGT1_309110, TGGT1_309020 and TGGT1_294380.

A search for tRNA-specific 2-thiolation enzymes in the *Toxoplasma* nuclear genome database (http://www.toxodb.org) using protein sequences of tRNA s^2^U34 thiouridylases in various species (see Materials and Methods) yielded 3 putative hits. These included TGGT1_309110 annotated as tRNA methyl transferase, TGGT1_309020 annotated as a PP-loop family protein, and TGGT1_294380 annotated as a PP-loop domain-containing protein. Phylogenetic analysis showed that TGGT1_309110 was more homologous to plants’ U34-tRNA thiouridylases MnmA, TGGT1_309020 had higher homology with cytosol Ncs6 of eukaryotes, while TGGT1_294380 had rather distant phylogenetic relationships with tRNA s^2^U34 thiouridylases in these species (**Figure 1A**). Further analysis indicated that TGGT1_294380 may belong to the TtcA/TtuA protein family involved in the 2-thiolation process of S^2^C32. Domain analysis using profile hidden Markov Models displayed that TGGT1_309110 contained one SufE domain (residues 519–589) and three tRNA_Me_trans domains (residues 812–952, 968-1140 and 1489-1560); TGGT1_309020 contained two PP-loop domains (residues 115-198 and 286-397); TGGT1_294380 contained one PP-loop domain (residues 1353-1528) and two aminotransferase class-V domains (residues 62-149 and 571-719) (**Figure 1B**). Based on phylogenetic analyses and domain prediction, we named TGGT1_309110 *MnmA*, TGGT1_309020 *Ncs6* and TGGT1_294380 *TtcA*.

### The nuclear tRNA thiouridylase *Tg*MnmA localizes to the apicoplast

**Figure 2:**
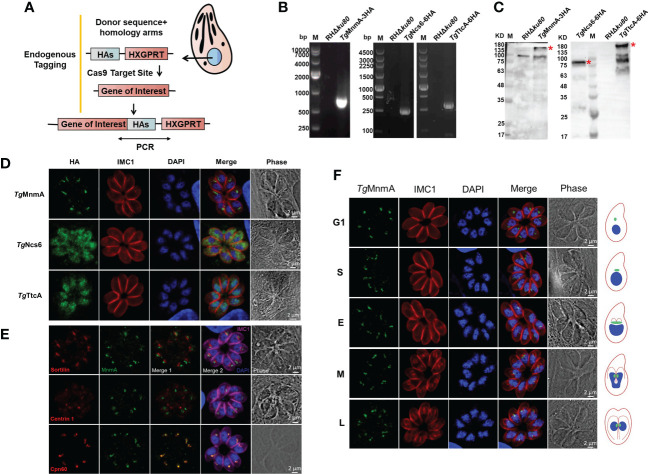
The tRNA thiouridylase *Tg*MnmA localizes to the apicoplast. **(A)** Schematic of the CRISPR/Cas9 strategy used to construct the endogenous HA-tagged tachyzoites. **(B)** PCR amplication of the HA-tag. The primers used were illustrated in Figure **(A)**. **(C)** Western blot identification of endogenously tagged proteins using rabbit anti-HA antibodies. The endogenously HA-tagged proteins of expected size are indicated by a red star. There is a nonspecific band of ~100kDa in the control lane. **(D)** Fluorescence microscopy of endogenously tagged *Tg*MnmA, *Tg*Ncs6 and *Tg*TtcA showing their intracellular locations. Parasites were labeled with rabbit anti-HA (green, indicating locations of endogenously tagged proteins) and mouse anti-IMC1 (red, a marker for the inner membrane complex), and DAPI (blue, nucleus). **(E)** Immunofluorescence co-localization analysis of *Tg*MnmA with different organelle makers. Parasites were labeled with rabbit anti-HA (green), mouse anti-sortilin (red, Golgi apparatus marker) or mouse anti-centrin 1 (red, centrosome marker) or anti-Cpn60 (red, apicoplast marker), rabbit anti-IMC1 (fuchsia, inner membrane complex 1), and DAPI (blue, nucleus). *Tg*MnmA is co-localized with Cpn60. **(F)** Expression dynamics and localization of *Tg*MnmA in the *Tg*MnmA-HA tagged cells. Parasites were labeled with rabbit anti-HA, mouse anti-IMC1 and DAPI. The right represents the schematic of *T. gondii* endodyogeny. The size, morphology and DNA intensity of cell nucleus (blue) combined with the extent of IMC1 (red) labeling of daughter scaffolds define the G1, S, M, early (E), intermediate (I), and late (L) stages of cytokinesis. Apicoplasts (green) showing different cell cycle stages are added. Scale bar, 2 μm.

In order to explore the potential functions of these proteins, we first determined their intracellular locations in *T. gondii* tachyzoites. Endogenously tagged cells were generated by inserting 3× or 6× hemagglutinin (HA) tags at the 3’ end of the coding sequence of each of the three genes in the RHΔ*Ku80* cells by CRISPR/CAS9 genome editing (**Figure 2A**). PCR yielded correct size bands for each cell (**Figure 2B**). Western blot showed that each protein in the endogenously HA-tagged cells migrated at the expected size using anti-HA antibodies, while the parental (RHΔ*ku80*) parasites did not (**Figure 2C**). Moreover, *Tg*TtcA has several bands with smaller molecular weight than the target size, which may be due to other processed forms or degradation products of the target protein. Immunofluorescence analysis (IFA) conducted with anti-HA antibodies demonstrated that these three proteins have distinct localizations in different subcellular compartments. *Tg*Ncs6 locates in the cytosol of the parasite, *Tg*TtcA locates in both the cytosol and nucleus, while *Tg*MnmA localizes to the juxtanuclear region (**Figure 2D**). To further clarify the localization of *Tg*MnmA, we conducted co-localization analysis experiments with antibodies recognizing different marker proteins including apicoplast marker (Cpn60), centrosome marker (Centrin 1), and Golgi marker (Sortilin), and found *Tg*MnmA colocalized with Cpn60, indicating that it is a nucleus-encoded apicoplast protein (**Figure 2E**). The apicoplast localization of *Tg*MnmA intrigued us because this characteristic plastid, found in most Apicomplexan parasites, is an ideal drug target in the treatment of toxoplasmosis. We found that the protein *Tg*MnmA is constitutively expressed during the cell cycle (**Figure 2F**), which is consistent with its mRNA transcription pattern (**Figure S1**) (Behnke et al., 2010). These results collectively suggest that *Tg*MnmA may play an important role in the asexual cycle of *T. gondii*.

### *Tg*MnmA is important for the parasite lytic growth

**Figure 3:**
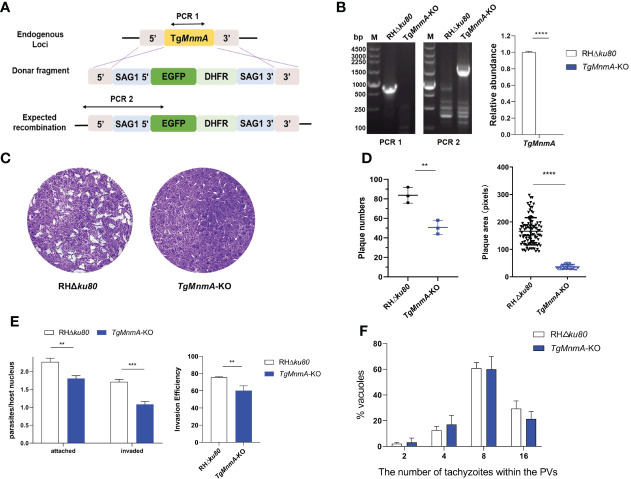
*Tg*MnmA is necessary for the lytic cycle of *T. gondii*. **(A)** Strategy of *TgMnmA*-KO construction. **(B)** Diagnostic PCR (left) and qRT-PCR (right) on representative clones P values: ****≤0.0001. The primers used for PCR are illustrated in Figure **(A)**. **(C)** Images of plaques formed by RHΔ*ku80* and *TgMnmA*-KO cells on HFF monolayers at day 7 with 200 parasites per monolayer. **(D)** Quantification of plaque numbers (left) and area sizes (right) of plaques formed by RHΔ*ku80* and *TgMnmA*-KO. P values: ** ≤ 0.01, **** ≤ 0.0001. **(E)** Tachyzoites were allowed to invade HFF cells for 30 min and then fixed, and extracellular parasites and total parasites were stained consecutively. The left panel shows attached and invaded parasite numbers of RHΔ*ku80* and *TgMnmA*-KO. The right panel shows invasion efficiency of tachyzoites representing invaded parasites/total parasites. P values: ** ≤ 0.01, ***≤0.001, **** ≤ 0.0001. **(F)** Ratios of parasite numbers to vacuoles are shown. Parasites within vacuoles were quantified after 24 h grown in HFFs.

To investigate the functions of *TgMnmA* on the lytic cycle biology of *T. gondii*, we generated *TgMnmA*-KO using the parental RHΔ*ku80* cells as described in Materials and Methods (**Figure 3A**). The deletion of *TgMnmA* was confirmed by both PCR and quantitative reverse transcription-PCR (qRT-PCR) (**Figure 3B**). We then performed plaque assays using *TgMnmA*-KO and RHΔ*ku80* cells to examine the effect of *Tg*MnmA on parasite growth *in vitro*, and measured the mean plaque number and size. A significant reduction was observed in both the number and size of plaques formed by *TgMnmA*-KO parasites compared to the parental RHΔ*ku80* cells (**Figures 3C, D**). Importantly, to confirm this phenotype, we constructed a *TgMnmA* complementary strain called *TgMnmA*-COMP, by complementing its full-length coding sequence combined with 3×HA-tag to the original locus, which was identified by PCR, western blot and IFA. We found that the complement of *TgMnmA* could rescue the phenotypic defects of the *TgMnmA*-KO parasite (**Figure S2**). To determine the effect of the knockout on parasite adhesion and invasion, a green-red adhesion/invasion assay was conducted, and we found that the number of parasites that adhered to and invaded host cells was significantly reduced, and the invasion efficiency was also significantly lower than that of RHΔ*ku80* (**Figure 3E**). Next, parasite replication was examined, and compared with RHΔ*ku80*, the replication rate of *TgMnmA*-KO was statistically insignificant, but showed a certain degree of reduction (**Figure 3F**). The results above demonstrate that *TgMnmA* plays an important role in lytic parasite growth of *Toxoplasma in vitro*.

### *Tg*MnmA specifically contributes to apicoplast biogenesis and probably its genome transcription

**Figure 4:**
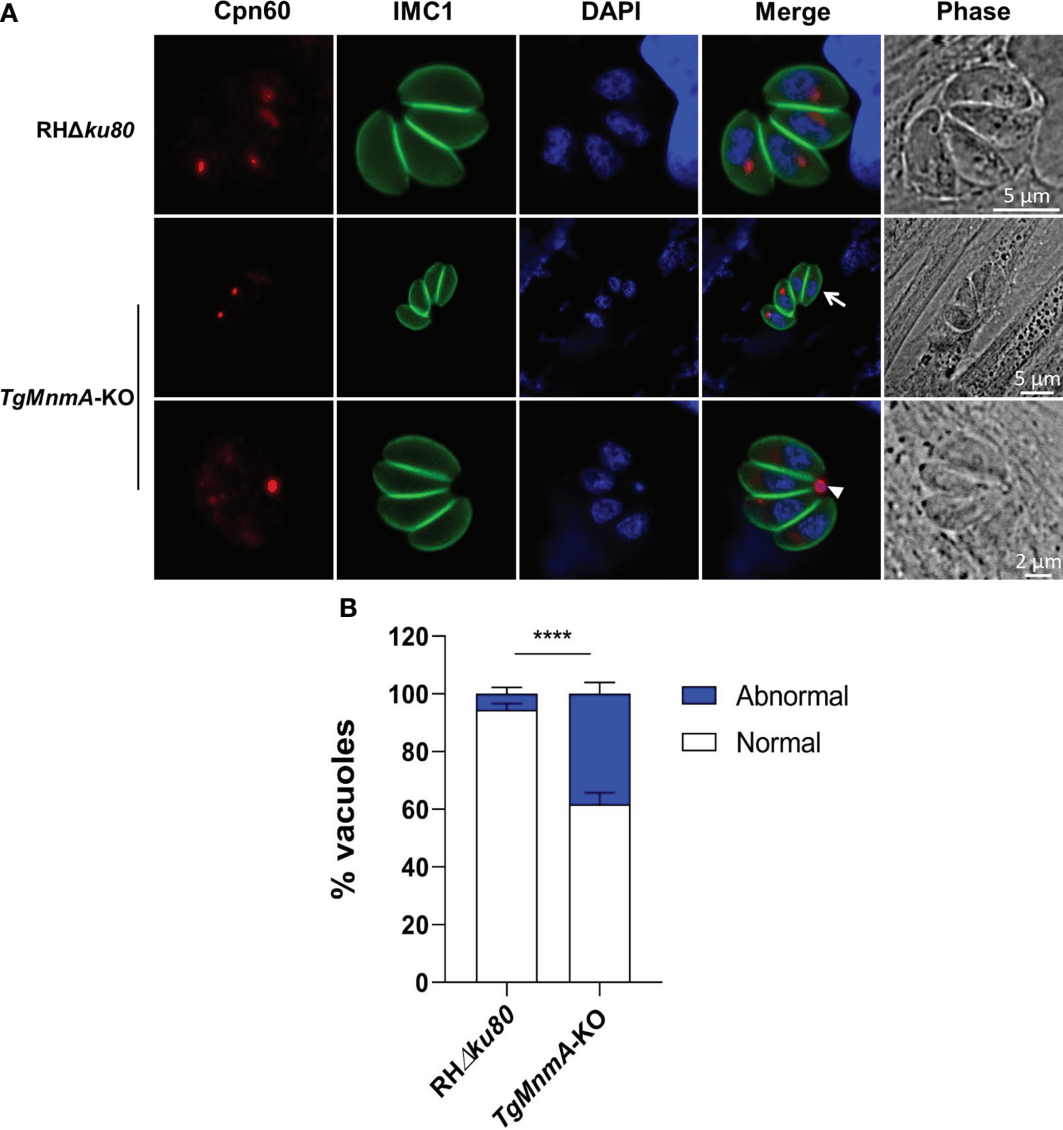
*Tg*MnmA is important for the apicoplast biogenesis. **(A)** Fluorescence microscopy of the apicoplast in 4-cell vacuoles of parental and *Tg*MnmA-KO parasites. Parasites were labeled with mouse anti-Cpn60 (green, apicoplast marker), rabbit anti-IMC1 (green, inner membrane complex 1), and DAPI (blue, nucleus). The arrow points to tachyzoites with no apicoplast, and the arrowhead, the apicoplast protein CPN60 was located at the residual body. Scale bar as shown in the Figure. **(B)** Quantification of the apicoplast in the parental RHΔ*ku80* and *Tg*MnmA-KO tachyzoites. Apicoplasts in the vacuoles with four tachyzoites each were counted at 16 h postinfection for each strain (n=100). Data represent mean ± SD (n = 9 replicates combined from n = 3 trials). Statistical significance was analyzed with an unpaired t test, p values: **** ≤ 0.0001.

The specific localization of *Tg*MnmA to apicoplast led us to explore the effect of *Tg*MnmA deficiency on apicoplast biogenesis. In the case of *TgMnmA* deletion, not all parasites in the parasitophorous vacuoles (PVs) were stained positive for Cpn60, an apicoplast lumen protein. *TgMnmA*-KO parasites in 4-cell vacuoles showed only 3, 2, 1, or even 0 apicoplast(s), and Cpn60 was located in the residual body in the PV rather than in the apicoplast cavity of the parasite (**Figure 4A**). Scoring of 100 4-cell PVs revealed that the proportion of apicoplast loss increased from 6% of the parental RHΔ*ku80* cells to 38% of *TgMnmA*-KO, confirming that *TgMnmA*-KO leads to apicoplast disappearance (**Figure 4B**). The effects of *TgMnmA*-KO on other organelles were also investigated. *TgMnmA*-KO seemed to have no observable effect on the centrosome, Golgi apparatus, rhoptry and dense granule (**Figure S3**). These data collectively show that *Tg*MnmA specifically contributes to the apicoplast biogenesis of *T. gondii*.

**Figure 5:**
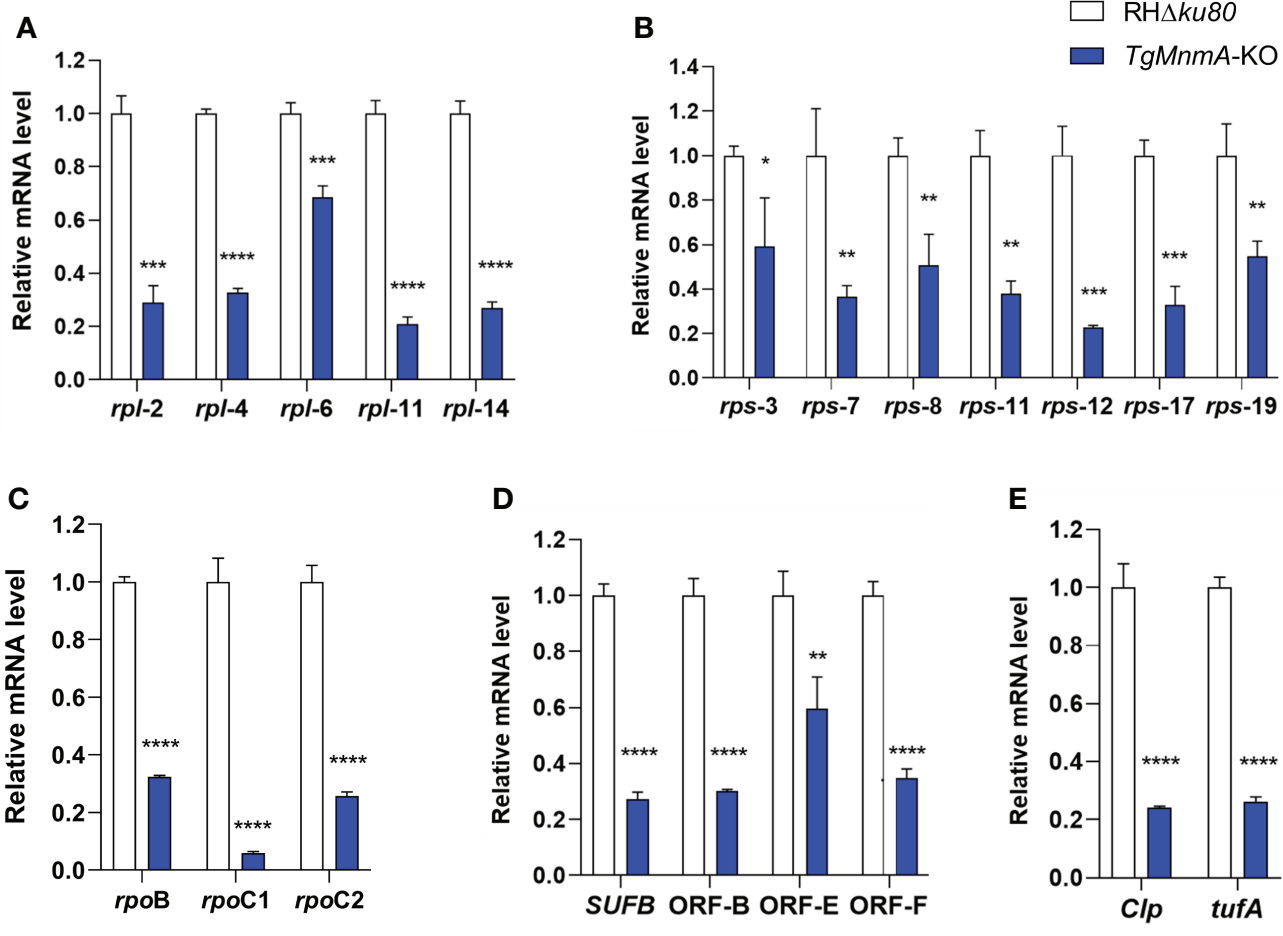
Knockout of *TgMnmA* affects *T. gondii* apicoplast genome transcription. mRNA quantification by qRT-PCR is shown for the large subunits of rRNA **(A)**, small subunits of rRNA **(B)**, RNA polymerases **(C)** and other apicoplast-encoded proteins **(D, E)** in RHΔ*ku80* and *TgMnmA*-KO grown in HFF monolayers for 24 h. Data represent means of three independent experiments, each done with triplicates. Error bars show the SEM. The statistical analysis was performed by 2^−ΔΔCt^ method in Excel 2017 and unpaired t test in GraphPad Prism 8. p values: * ≤ 0.05, ** ≤ 0.01, *** ≤ 0.001, **** ≤ 0.0001.

We next investigated *Tg*MnmA on gene transcription in apicoplast using qRT-PCR with cDNA from *T. gondii* tachyzoites grown in HFF monolayers for 24 h. The relative mRNA levels of apicoplast genes reduced from 31% to 94% in *TgMnmA*-KO compared to the parental RHΔ*ku80*. These included the large and small rRNA genes (**Figures 5A, B**), the RNA polymerases (*rpoB*, *rpoC1* and *rpoC2*) (**Figure 5C**), other important genes such as *Clp*, *tufa* and *SUFB*, and ORFs of unknown function (**Figures 5D, E**). These data collectively demonstrate that *Tg*MnmA is probably required for expression of the apicoplast genome in *T. gondii* tachyzoites.

### ATc inhibits apicoplast replication and *Tg*MnmA expression

**Figure 6:**
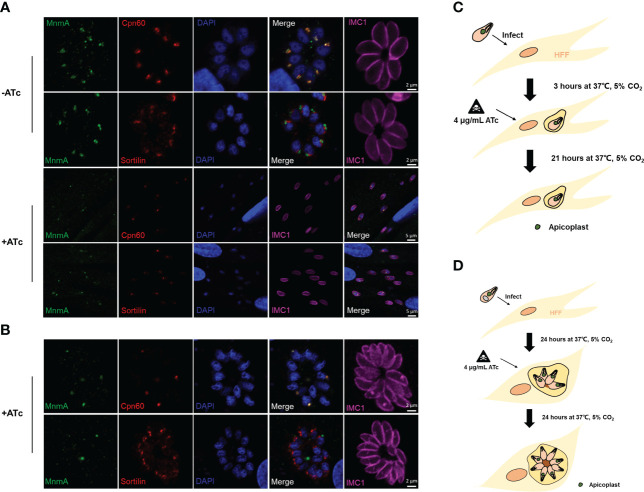
ATc treatment inhibits apicoplast replication and *Tg*MnmA protein expression. Effects of ATc on apicoplast biogenesis and *Tg*MnmA protein expression were detected by IFA after treating the *Tg*MnmA-3HA tachyzoites with or without ATc for different times. **(A)** Tachyzoites were used to infect HFF cells for 3 h and then treated with or without ATc for 21 h ATc treatment had no significant effect on the already-existing apicoplasts and *Tg*MnmA protein expression, but it inhibited parasite replication in the vacuole. **(B)** Tachyzoites infected HFF cells for 24 h and then were treated with ATc for an additional 24 h. The ATc-treated parasites continued 2 to 4 cycles of endodyogeny, but the apicoplast replication was inhibited as shown by lack of the apicoplast in some cells, and the *Tg*MnmA protein is below the detectable levels in the parasite with no apicoplast. Cells are stained with rabbit anti-HA (green, indicating *Tg*MnmA), mouse anti-Cpn60 (green, apicoplast marker) or mouse anti-Sortilin (green, Golgi apparatus marker), rabbit anti-IMC1 (fuchsia, inner membrane complex 1), and DAPI (blue, nucleus). **(C** ,**D)** Schematic presentation of the effect of ATc treatment.

It has been shown that anhydrotetracycline (ATc) blocks replication and transcription of the apicoplast genome, resulting in loss of the *T. gondii* apicoplast (Dahl et al., 2006; Wiesner et al., 2008). The expression of apicoplast marker Cpn60 and the target *Tg*MnmA was investigated by IFA after the *Tg*MnmA-3HA cells were treated with or without ATc upon host cell entry for 3h or 24h. At 3h PI, ATc treatment had no obvious effect on the existing apicoplast and *Tg*MnmA protein, but it inhibited parasite’s replication in the vacuole (**Figures 6A, C**). Once intracellularly located after 24 h incubation of tachyzoites with HFF cells, the parasites continued 2 to 4 cycles of endodyogeny even after ATc treatment although their apicoplast replication was inhibited. These were demonstrated by some apicoplast-less progenies and undetectable *Tg*MnmA protein (**Figures 6B, D**). These results were verified by Western blot (**Figure S4**). Furthermore, in both cases, ATc drug treatment did not significantly affect the Golgi apparatus as indicated in the Sortilin panels of **Figure 6**. These results unequivocally indicate that ATc treatment prior to tachyzoite replication inhibits apicoplast replication of the single tachyzoite, resulting in replication inability. However, once tachyzoites have undergone endodyogeny, the parasites can continue to proliferate even in the presence of ATc, but replication of the apicoplast is inhibited resulting in apicoplast-less progenies and no *Tg*MnmA proteins.

### Mice survive after infection with 10 *Tg*MnmA-KO tachyzoites

**Figure 7:**
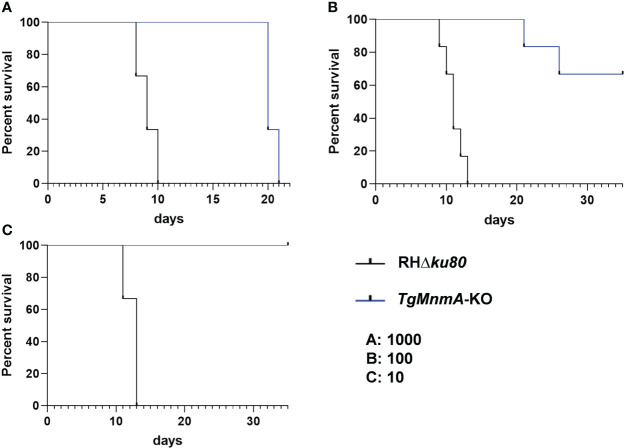
Survival of mice is monitored after infection with variable doses of RHΔ*ku80* and *Tg*MnmA-KO tachyzoites. ICR mice were infected intraperitoneally with 1000 **(A)**, 100 **(B)** and 10 **(C)** tachyzoites of RHΔ*ku80* and *Tg*MnmA-KO tachyzoites (Six female mice per group), and their survival was monitored twice daily for over 35 days.

To determine whether *Tg*MnmA is required for survival *in vivo*, ICR mice were intraperitoneally infected with variable doses of *TgMnmA*-KO or RHΔ*ku80* tachyzoites. Mice injected with 1000 RHΔ*ku80* tachyzoites died within 10 days, whereas mice infected with 1000 *TgMnmA*-KO tachyzoites lived to at least 20 days post-infection (PI) (**Figure 7A**). In the groups of mice receiving 100 tachyzoites, all mice given RHΔ*ku80* succumbed to lethal toxoplasmosis within 13 days of infection. In contrast, among the mice injected with *TgMnmA*-KO, one mouse died at day 21 PI, another one died at day 26 PI, and 4 mice remained alive 35 days PI, with a mortality rate of only 33.3% (**Figure 7B**). Further, mice infected with 10 RHΔ*ku80* tachyzoites also died within 13 days, conversely, all mice inoculated with 10 *TgMnmA*-KO tachyzoites remained alive 35 days PI (**Figure 7C**). Importantly, we found that the complement of *TgMnmA* could restore the wild-type phenotype (**Figure S5**). Moreover, to confirm the survival of mice from parasitic exposure, antibodies to *T. gondii* were detected in sera of these mice collected at 20 days PI. These data clearly show that mice could survive longer after infection with *TgMnmA*-KO tachyzoites than those with wild-type RHΔ*ku80* tachyzoites.

## Discussion

Sulfur is an essential element for a variety of cellular constituents in all living organisms. The derivatives of s^2^U, s^4^U, s^2^C, and ms^2^A are common sulfur-containing nucleotides (Zheng et al., 2021). Previous studies have shown that these modifications make translation much more accurate and efficient, such as proper recognition of the codons in mRNA or stabilization of tRNA structure (Liu et al., 2016).The biosynthesis of tRNA sulfur modification involves the “sulfur transport process” initiated by cysteine ​​desulfurase and the “integration process” of tRNA involved by the modification enzymes (Laxman et al., 2013). Compared with sulfur-modified nucleotides at six positions (8, 9, 32, 33, 37, and 54) in tRNAs, the U34 site is prone to occur 2-thiolation modification (Cavuzic and Liu, 2017). In eukaryotes, thiouridylases have been found in both the cytoplasm and the mitochondrion, and are involved in s^2^U34 modification of glutamate, glutamine, and lysine tRNAs. Recently, mnm^5^s^2^U modification in chloroplast tRNAs has been reported (Liu et al., 2020), while the modification enzyme responsible for s^2^U modification has not been identified. Among the three purported *T. gondii* tRNA s^2^U34 thiouridylases, one was confirmed in the cytoplasm and another one in the apicoplast by endogenous tagging mediated by CRISPR/Cas9 technology, and they were named as *Ncs6* and *MnmA*, respectively. However, we didn’t identify the 2-thiouridylase specifically located in the mitochondrion, participating in the formation of the s^2^U34 derivative. We speculate that probably tRNAs in the mitochondrion are very likely imported from the cytoplasm in their 2-thiolated form, since it has been found that there is no mitochondrion-encoded tRNA gene in *T. gondii*, and its translation is compensated by the import of cytosolic tRNAs encoded by the nucleus and very likely imported in their aminoacylated form in Apicomplexa (Pino et al., 2010).

In this study, deletion of *TgMnmA* led to a significant decrease in the number and area of plaques. Further, *TgMnmA*-KO parasites showed a significantly attenuated phenotype in mice. Altogether they domonstrate that *Tg*MnmA plays an important role in *T. gondii* not only *in vitro* but also *in vivo*. It has been reported that tRNA modification is essential for the growth and development of malaria parasites. 28 tRNA modifications in asexual IDC of *Plasmodium falciparum* were identified, among them 22 were observed with a synchronized increase from ring to trophozoite stage. It was found that s^2^U34 modification plays necessary roles during various stages of malaria parasites (Ng et al., 2018). In the ring stage, s^2^U derivatives formed by the high level of s^2^U34 modification may serve as the modification precursors for the subsequent growth stage. In the ring-to-trophozoite transition, s^2^U34 modification and other tRNA modifications work together to ensure the accurate and efficient synthesis of proteins. In the trophozoite stage, the dithio modification of the wobble site U34 enhances the specific recognition of “codon-anticodon”, resulting in selective gene expression. However, the true roles of different tRNA modification, the distribution and location of tRNA modification, and the modifying enzymes involved in apicomplexan parasites remain unknown. U-ending anticodons of tRNAs are almost always modified to ensure accurate decoding (Agris, 2004; Juhling et al., 2009), and our findings first clarify the key role of the thiouridylase *Tg*MnmA involved in apicoplast s^2^U34 modification in the *in vitro* growth and *in vivo* survival of *T. gondii* tachyzoites, and provide an important basis for the future research.

The intracellular growth process of *T. gondii* includes adhesion and invasion of host cells, replication in the PV and egress from host cells. Various assays using *TgMnmA*-KO were carried out to tease out *Tg*MnmA’s function involved in these processes. It turned out that *TgMnmA*-KO significantly decreased adhesion and invasion, but had little effect on replication at 24 h. Quantification of the apicoplast-resident protein (Cpn60) showed loss of the apicoplast in a fraction of the population. We speculated that abnormalities of the apicoplast cause tachyzoites’ defects in adhesion and invasion, and that the underlying mechanism needs to be worked out in the future studies. While not all the parasites within a single PV have the apicoplast, the apicoplasts in some parasites are capable of driving the parasites without the apicoplast in the same PV to undergo one more round of complete IDC before death (Kennedy et al., 2019), demonstrating little effect on replication at 24 h. One RH parasite can kill ICR mice within 15 days. Mice survive from infection with 10 tachyzoites of the *TgMnmA*-KO strain as tachyzoites without apicoplasts may do not survive long after 24 h. The attenuated phenotype is probably due to the significantly reduced ability of parasite adhesion and invasion and/or other mechanisms, as tRNA thiouridylases have a variety of vital biological functions.

Owing to the prokaryotic origin of plastids, the apicoplast DNA replication and protein translation apparatuses are inherently bacterium-like in nature (Köhler et al., 1997). In particular, bacterial antibiotics, such as tetracyclines, have been readily repurposed to treat toxoplasmosis (Neville et al., 2015). In addition, it was confirmed that tetracyclines specifically target the apicoplast rather than the mitochondria (Dahl et al., 2006; Wiesner et al., 2008). In this study, treatment of *Tg*MnmA-3HA tagged parasites with high doses of ATc (4 μg/mL) at 24 h PI resulted in some apicoplast-less parasites along with apicoplast positive cells within a single PV. The treatment did not interfere with parasite division and rosette formation, which is consistent with the previous research (Jacot et al., 2013). However, when this treatment took place at 3 h PI, no parasite replication occurred. These intriguing results indicate that ATc could block parasite replication when there is only one tachyzoite in a PV. Nevertheless, when there are two or more tachyzoites, the existing apicoplasts are capable of driving parasites to undergo one more round of complete IDC before death. This phenomenon is worth further exploration. Furthermore, in the absence of apicoplast, the expression of nucleus-encoded apicoplast proteins such as Cpn60 and *Tg*MnmA were also blocked, which confirms the previous report that ATc treatment blocks the apicoplast protein biosynthesis (Dahl et al., 2006).

*Tg*MnmA is considered to be a conserved tRNA modifying enzyme that modifies 2-thiouridine at the wobble position of apicoplast tRNA^Gln^, tRNA^Glu^ and tRNA^Lys^. We observed that deletion of *TgMnmA* results in the loss of the apicoplast in a fraction of the parasites, consistent with the fact that *mtu1* knock-out zebrafish has decreased mitochondrial numbers in hair cells of inner ear (Zhang et al., 2018), and *mtu1* is a homolog responsible for mitochondrial s^2^U modification (Yan and Guan, 2004; Armengod et al., 2014). The primary defect in the *TgMnmA*-KO is expected to be the deficient biosynthesis of s^2^U at U34 of tRNA^Lys^, tRNA^Glu^ and tRNA^Gln^. It is anticipated that the failure in tRNA metabolism caused by *TgMnmA* deletion perturbes the apicoplast protein synthesis and function. Using qRT-PCR, we found that the absence of *TgMnmA* significantly reduced the transcriptional levels of the apicoplast genome including those of the large and small rRNA, the RNA polymerases and other important proteins. These data suggested that inactivation of *TgMnmA* may result in reduced levels of s^2^U modification in apicoplast tRNAs, leading to occurrence of translational errors in the apicoplast. Abnormal apicoplast translation may further result in aberrant ribosome biogenesis, reduced levels of apicoplast-encoded proteins, and impaired apicoplast-encoded RNA polymerase activity, similar to the the functional defect of PDDOL in chloroplast tRNAs by reduced levels of mnm^5^s^2^U modification (Liu et al., 2020). while we have shown an important role for *Tg*MnmA in the apicoplast biogenesis, more evidence is needed to ascertain if it is directly involved in its genome transcription in the future research.

In summary, *Tg*MnmA is a nucleus-encoded apicplast tRNA sulfur-modifying enzyme. The absence of *TgMnmA* results in the inhibition of intracellular growth of the parasite. *Tg*MnmA plays a critical role in maintaining the function of the apicoplast. These fndings demonstrate the critical role of *Tg*MnmA in apicoplast biogenesis and parasite growth and indicate this protein as an important candidate for therapeutic intervention.

## Data availability statement

The original data presented in the study are included in the article/**Supplementary Material**. Further inquiries can be directed to the corresponding author.

## Ethics statement

All animal experiments were conducted in accordance with the requirements of the Animal Ethics Procedures, approved by the Experimental Animal Ethics Committee, Zhejiang University. This research followed the guidance of the Guiding Principles for the Care and Use of Laboratory Animals of Zhejiang University (ZJU20170177).

## Author contributions

Conceptualization, YMY and AD. Methodology design, YMY, ML, and LC. Formal analysis, YMY, ML, XZ, and YY. Investigation, YMY, ML, XC, LC, and KS. Writing – original draft, YMY and ML. Writing – review and editing, AD, YMY, CY, and GM. Visualization, YMY and ML. Supervision, AD, YMY, and YY. Project administration, YMY, ML, XC, LC, MZ, and KS. Funding acquisition, AD, YMY, and YY. All authors contributed to the article and approved the submitted version.

## Funding

This project was funded by the National Natural Science Foundation of China (grant No. 31672543), the Natural Science Foundation of Zhejiang province, China (grant No. LQ21C180002), Zhejiang Province “Sannongliufang” Science and Technology Cooperation Project (grant No. 2020SNLF007)

## Acknowledgments

We are thankful to Yunqin Li (Analysis center of Agrobiology and environmental science, Zhejiang University) for technical assistance on laser confocal microscopy. We would like to acknowledge The Experimental Teaching Center, College of Animal Sciences, Zhejiang University for the assistance with qRT-PCR.

## Conflict of interest

The authors declare that the research was conducted in the absence of any commercial or financial relationships that could be construed as a potential conflict of interest.

## Publisher’s note

All claims expressed in this article are solely those of the authors and do not necessarily represent those of their affiliated organizations, or those of the publisher, the editors and the reviewers. Any product that may be evaluated in this article, or claim that may be made by its manufacturer, is not guaranteed or endorsed by the publisher.

## Supplementary material

Supplementary Figure 1
*Tg*MnmA relative expression levels throughout the cell cycle as reported in reference 19.

Supplementary Figure 2 Detection of plaques formed by RHΔku80, *TgMnmA*-KO and *TgMnmA*-COMP strains. **(A)** Images of plaques formed by RHΔku80, *TgMnmA*-KO and *TgMnmA*-COMP on HFF monolayers at day 7 with 200 parasites per monolayer. Quantification of plaque numbers **(B)** and area sizes **(C)** of plaques formed by RHΔku80, *TgMnmA*-KO and *TgMnmA*-COMP. P values: * ≤ 0.05, **** ≤ 0.0001.

Supplementary Figure 3 The positioning and integrity of the organelles were examined in intracellular RHΔ*ku80* and *TgMnmA*-KO parasites. The rhoptries (α-ROP14), dense granules (α-GRA16), Golgi apparatus (α-Sortilin) and centrosome (α-Centrin 1) do not present any visible defect. The parasites were co-stained with α-IMC1 (pellicle) and DAPI (nucleus). Scale bars, 2 µm.

Supplementary Figure 4 Western blot showing expression of *Tg*MnmA-3HA 24 hours ± ATc. Lysates of Intracellular tachyzoites were probed with rabbit anti-HA monoclonal antibody (upper panel). SAG1 was used as loading control (bottom panel).

Supplementary Figure 5 ICR mice were infected intraperitoneally with 10 tachyzoites of RHΔku80, *TgMnmA*-KO and *TgMnmA*-COMP strains (Six female mice per group), and their survival was monitored twice daily for over 35 days.

Supplementary File 6 Amino acid sequences and the associated accession numbers used to generate phylogenetic tree.

Supplementary Table S1 Gene contents of the Toxoplasma gondii apicoplast genome.

Supplementary Table S2 Sequences for primers used in this study.
