# Supplementary material for: The first apicoplast tRNA thiouridylase plays a vital role in the growth of Toxoplasma gondii
Source: Front Cell Infect Microbiol. 2022 Aug 15;12:947039. doi: 10.3389/fcimb.2022.947039 (PMC9420914; doi:10.3389/fcimb.2022.947039)
Supplement: Supplementary File 6 — Amino acid sequences and the associated accession numbers used to generate phylogenetic tree. [file DataSheet_1.docx]

>NP_060476.2 mitochondrial tRNA-specific 2-thiouridylase 1 isoform a [Homo sapiens]

MQALRHVVCALSGGVDSAVAALLLRRRGYQVTGVFMKNWDSLDEHGVCTADKDCEDAYRVCQILDIPFHQVSYVKEYWNDVFSDFLNEYEKGRTPNPDIVCNKHIKFSCFFHYAVDNLGADAIATGHYARTSLEDEEVFEQKHVKKPEGLFRNRFEVRNAVKLLQAADSFKDQTFFLSQVSQDALRRTIFPLGGLTKEFVKKIAAENRLHHVLQKKESMGMCFIGKRNFEHFLLQYLQPRPGHFISIEDNKVLGTHKGWFLYTLGQRANIGGLREPWYVVEKDSVKGDVFVAPRTDHPALYRDLLRTSRVHWIAEEPPAALVRDKMMECHFRFRHQMALVPCVLTLNQDGTVWVTAVQAVRALATGQFAVFYKGDECLGSGKILRLGPSAYTLQKGQRRAGMATESPSDSPEDGPGLSPLL

>NP_001129348.1 mitochondrial tRNA-specific 2-thiouridylase 1 [Rattus norvegicus]

MSALRHVVCALSGGVDSAVAALLLRRRGYQVTGVFMKNWDSLDEQGICAADKDCEDAYKVCQILDIPFHQVSYVKEYWNDVFSDFLNEYEKGRTPNPDISCNKHIKFSCFHHYAVDNLGADAVATGHYARTSLEDEEVFEQKHTKRPDGLFRNRFEVRNPVKLLQAADSFKDQTFFLSQVSQDALRRTIFPLGELTKDFVKKIAAENRLHHVLQKKESMGICFIGKRNLEHFLLQYLQPRPGKFISIEDNRVLGTHKGWFLYTLGQRAKISGLSEPWYVVEKDGTKGDVLVAPRVDHPALYRDLLRTNRVHWIAEEPPAALVRDKMMECHFRFRHQMALVPCVLTLNQDGTVWVTAVKAVRGLALGQFAVFYKGEECLGSGKILRLGPSAYTLQKGKNRTRVAPEVSSDSPGLHPTS

>NP_082339.1 mitochondrial tRNA-specific 2-thiouridylase 1 [Mus musculus]

MSALRHVVCALSGGVDSAVAALLLRRRGYQVTGVFMKNWDSLDEQGVCAADKDCEDAYKVCQILDIPFHQVSYVKEYWNDVFSDFLNEYEKGRTPNPDINCNKHIKFSCFYHYAVDNLGADAVATGHYARTSLEDEEVFEQKHTKKPDGLFRNRFEVRNPVKLLQAADSFKDQTFFLSQVSQDALRRTIFPLGELTKDFVKKIAAENSLHHVLQKRESMGICFIGKRNLEHFLLQYLQPRPGKFVSIEDNTVLGTHKGWFLYTLGQRAKISGLREPWYVVEKDGTKGDVLVAPRVDHPALYRDLLRTNRVHWIAEEPPAALVRDKMMECHFRFRHQMALVPCVLTLNQDGTVWVTAVKAVRGLALGQFAVFYKGEECLGSGKILRLGPSAYTLQKGKNRTRVAPEASSDSPGLHPTS

>NP_652639.1 mitochondrial tRNA-specific 2-thiouridylase 1 [Drosophila melanogaster]

MIRNVVVGVSGGVDSAVSAHLLAEQGFKVLGVFMRNWDEADEVGRCSGEADLKDAEWACRQLGVELRQVNYVREYWTAVFSQFLDDYQMGLTPNPDILCNRHIKFDLFHKHALENLGYDAVATGHYARNSLGNYLEGIASNNDARLLIPADTFKDQTFFLAGISRKALQRTMFPLGDFQKSQVKDLAKKIGFQRLAKKKESTGICFVGKRNFKDFIQEYITSKRGPFLDIDSGAVVGHHEGIHQWTVGQRCRLSSFLQPYFVARKEAASNTIYVASGHNHPALLSTHIAVDPPNWLCSKSQQILSDTGSLRCRFRFQHTKPLVDCQLSISPSNTFLVELDAPLRAITPGQYAVFYDDTACLGSARILSANPLKKKNAQTQQAQAANLVS

>XP_011211827.1 mitochondrial tRNA-specific 2-thiouridylase 1 [Bactrocera dorsalis]

MFRNVVIGISGGVDSAISALLLKQKGYNVIGAFMKNWDEFDEMGACSGEQDYRDAEYTCQKLDIELHTVNYVKEYWNSVFSAFLEDYQNGLTPNPDILCNKYIKFDLFYKYATEQLQCDAIATGHYAKTSFGNFLQLYNANGAVHLLIPRDTFKDQTFFLSGIQRHTLSRTMFPLGDALKQEIKALARECGLERLAQKKESTGICFVGNRDFKQFIKEYIQPRTGHFVDIDNGKIVGTHEGIHTWTIGQRCRLASYLRPYFVAQKDVANNVIYVASGHEHPALYSDVIHAGAPNWLCNDPLSAGADVAGQQLRCRFRFQHTKPLVDCVVENSAKETGGVTITLDKPLRALTPGQYAVFYSETACLGSARIVSACRRAEDRLAEVEILKPS

>XP_012158526.1 mitochondrial tRNA-specific 2-thiouridylase 1 isoform X1 [Ceratitis capitata]

MFRNIVIGVSGGVDSAISALLLKQKGYNVIGLFMKNWDEFDEMGACSGEQDYRDAEFTCQKLDIELHMVNYVKEYWNSVFRCKLGFPLPVSFFFRFFFSYISAFLEDYQNGLTPNPDILCNKYIKFDLFYKYARGHLHGDAIATGHYAKTSFGNFLEHYNANGDVNLLIPRDTFKDQTFFLSGIQRHTLSRTMFPLGDSLKSDVKLLARKCGLERLAHKKESTGICFVGNRDFKQFIKEYIQPRPGNFVDIDSGHIVGTHGGIHEWTIGQRCRLASYLRPYFVAKKDVANNTIYVASGHEHPSLYSDIINASAPNWLCNDPLGAGTSELRCRFRFQHTKPLVDCTVKRTKGNTSGVVITLDKPLRALTPGQYAVFYSGSACLGSARIISASAKVAESENKLARVGVEERVRSMKQR

>NP_502120.1 putative mitochondrial tRNA-specific 2-thiouridylase 1 [Caenorhabditis elegans]

MPRVVIGMSGGVDSAVSAFLLKKRGFDVIGLHMINWDVQEEGTSHCPRSKDESDARNVCDRLNIPFHTVNFVKEYWNDVFLKFLENYKNGRTTVPDIDCNQSIKFDVFHKIAREKFNADFIATGHYATTNFGDFQQNAKDSDEIRLFSGKDPLKDQTFFLCTVNQEQLKRAMFPLGSLQKSEVKRIAEEQGFQEVAKKPESMGICFIGKKKRFSDFLDEYIEPKPGRILLKNGSEIGNHHGIHQFTIGKRINGKYLEARSHLGFFVSHIHSDTGDIIACEGSHHPDLYASRFLINHPKWIRTFDPFNRISSNNFLCRIQRTHPPIPCVAEKQEQFLSVIPRLALRATAPGQMCVFYNTKNECLGGGEIMNIQETL

>NP_415651.4 tRNA-specific 2-thiouridylase [Escherichia coli str. K-12 substr. MG1655]

MSETAKKVIVGMSGGVDSSVSAWLLQQQGYQVEGLFMKNWEEDDGEEYCTAAADLADAQAVCDKLGIELHTVNFAAEYWDNVFELFLAEYKAGRTPNPDILCNKEIKFKAFLEFAAEDLGADYIATGHYVRRADVDGKSRLLRGLDSNKDQSYFLYTLSHEQIAQSLFPVGELEKPQVRKIAEDLGLVTAKKKDSTGICFIGERKFREFLGRYLPAQPGKIITVDGDEIGEHQGLMYHTLGQRKGLGIGGTKEGTEEPWYVVDKDVENNILVVAQGHEHPRLMSVGLIAQQLHWVDREPFTGTMRCTVKTRYRQTDIPCTVKALDDDRIEVIFDEPVAAVTPGQSAVFYNGEVCLGGGIIEQRLPLPV

>WP_003459581.1 tRNA 2-thiouridine(34) synthase MnmA [Clostridium perfringens]

MTKKKVVIGMSGGVDSSVAAYLLKEQGYDVIGVTMQIWQEDKEYEEREGGCCSLSAVDDARRVAQKLDIPFYVLNFRDSFKRNVIDYFVDEYIQGRTPNPCIACNKYLKFDELLQKAKGIGADYVATGHYAKIEERDGRFQLIRSKDDRKDQTYALYNLTQEQLEHTLMPCGEFTKDKIREIAKEIGLDVHNKKDSEEICFIPDNNHGRYICEAAPNKVRPGNFVDKYGNVLGKHKGIVYYTIGQRKGLGLALGRPVFVTDINPVTNTVVVGPEEDIFKTDLVCKDINFISIDKLEGPMEVEAKIRYSARPAKATISPMENGRVKVSFEDKQRAITKGQSVVFYKDDLVVGGGIIESLL

>WP_012353829.1 tRNA 2-thiouridine(34) synthase MnmA [Cupriavidus taiwanensis]

MSGAAKRVVVGMSGGVDSSVTAWLLKQQGYEVIGLFMKNWEDDDDSEYCSTRQDWLDVVSVADLIGVDVEAVNFAAEYKDRVFADFLREYSAGRTPNPDVLCNAEIKFKAFLDHAMSLGAETIATGHYARVRQNAAGRFELLKALDHTKDQSYFLHRLNQAQLSRTLFPLGEIPKTRVREIAAEIGLPNAKKKDSTGICFIGERPFRDFLNRYLPTKPGPMKTPEGKVVGEHIGLAFYTLGQRKGIGLGGSRDGNGDAWYVARKDMANNTLYVVQGHDHPWLLTPVLNASDLSWVAGEPPAAGAAMAAKTRYRQSDAACTVQAVDADALSLGFAEPQWAVTPGQSAVLYDGDICLGGGIIQ

>WP_013318094.1 tRNA 2-thiouridine(34) synthase MnmA [Dickeya dadantii]

MSDNSQKKVIVGMSGGVDSSVSAYLLQQQGYRVEGLFMKNWEEDDDTEYCSAATDLADAQAVCDKLGIELHTVNFAAEYWDNVFEHFLAEYRAGRTPNPDILCNKEIKFKAFLEFAAEDLGADYIATGHYVRRKDVDGKSRLLRGLDGNKDQSYFLYTLSHQQLAQSLFPVGELEKPQVREIAEQLDLATARKKDSTGICFIGERKFRDFLARYLPAQPGPILSVDDGKVMGEHQGLMYHTLGQRKGLGIGGVKEGGDDPWYVVDKDVANNVLYVAQGHEHPRLMSVGLIAQQLHWVDREPLTQPLRCAVKTRYRQADIPCLLTPLDADRIEVRFDEPVAAVTPGQSAVFYQGEACLGGGIIEERLRAAQ

>WP_012740641.1 tRNA 2-thiouridine(34) synthase MnmA [Lachnospira eligens]

MKKKVVVGMSGGVDSSVAAYLLKEQGYDVIGVTMQIWQDDATEAAENGCCGISAVDDARRVAGQLDIPYYVMNFKNEFKCNVIDYFVDEYKKGRTPNPCIACNRYVKWESLLKRSLEIGADYIATGHYARIHQLPNGRYTICNSVTARKDQTYALYNLTQEQLSHTLLPIGDYTKDQVREIASKIGITVAKKPDSMEICFIPDNDYAGFIKRETGYTSVPGNFIDVNGNVIGRHQGIIHYTVGQRKGLGLALGKPAFVVAIRPETNEVVIGDNSDVFSPKLYANNLNFMSIPSLEGEMRAKGKIRYSHEGAMCTIRMFDENTLECTFDEPVRAVTPGQALVLYDGDNVLGGGTII

>XP_015631825.1 tRNA-specific 2-thiouridylase MnmA [Oryza sativa Japonica Group]

MLRVVSTLPALRPLLAGSTLLLNARPLLRSRLTRRPFRAVSSSTASPSSSSSGARDFGGVDFGDERLLRCAAAGRAPLRVAVLVSGGVDSSVALRLLHAAGHRCTAFYLKIWFQEDFRNFWSECPWDDDLKYAQAVCDKIDVPLEVVHLSDEYWNHVVSHIINEYRSGRTPNPDVLCNTRIKFGAFLEAIENLGFDYIASGHYAHVVHPSPDDVEGPSVLQLSKDKVKDQTYFLSHLSQTQLRRLLFPLGCITKDEVRRLAAQMDLPNQDRKDSQGICFLGKVKFSEFVERQIGEMEGVLLEAETGDYLGTHRGFWFYTIGQRQGLRLPGGPWYVVEKGVQNNVVFVSRNYYSLDKRRRTFRVGSLNWFSNSGPTNNEQLKCKVRHSPEFHDCTVTQEQTSEHGVILVVRLSEDDQGLAAGQFAAFYRDNLCLGSGIILDSWDEMNFPVCARALEIARMEDKSRLGKPVKIMNLEHIVKSEKEAIEVA

>XP_037471941.1 tRNA-specific 2-thiouridylase MnmA-like, partial [Triticum dicoccoides]

LEAIENLGFDYIASGHYAHVVHPHVENAKEPSVLQLLKDEIKDQTYFLSHLSQPQLRRLLFPLGCITKDEVRSLAVQMDLPNQARKDSQGICFLGKVKFSEFVQRHIGEMEGILLEAETGDYLGMHRGFWFYTIGQRQGLRLSGGPWYVVEKDVQNNVVFVSRNYYSLDKRRRTFRVGSMNWFSDSGPAITARRHERRDEEIF

>XP_013655567.1 tRNA-specific 2-thiouridylase MnmA-like [Brassica napus]

MFSTVMKPLLSPSPSLFRLPPLSSSLSLFFRVFPFSSSSSSHFHSPHPITPLRSSRRNYTRHHASLSDHYLSCSVPEKPLRVAVLLSGGVDSSVALRLLHAAGHSCTAFYLKIWFQEGFENFWNQCPWEDDLKYAKHVCEQVGVPLEVVHLTDEYWVRVVSYIIEEYKCGRTPNPDVLCNTRIKFGAFMDAISDMEYDYVASGHYAKVAHPAAGGPSVLELSQDMVKDQTYFLSHLSQTQLKRLLFPLGCVKKEEVRKLATQFDLPNKDRKDSQGICFLGKIKFSDFVGTHIGEKEGIILEAETGDFLGNHKGFWFYTIGQRQGLRLPGGPWYVVEKDTKNNVVFVSRDYYSIDKRRRVFRAGSLRWLSGKPLVNISQLRCKVRHGPGFYSCSFEMEAEGDVAVVHLDEDDQGLAAGQFAAFYEGTICIGSGVILESWDDQCFPVCAKALQLAAVEDKTKLGKPVKIMTMPVTTSVEAEPGESSGEERLVNA

>XP_025695650.1 tRNA-specific 2-thiouridylase MnmA [Arachis hypogaea]

MMLRVAVRWSCWNPMTSLPLPTIRFPRRPRLLRFPASNLLSPLSASAALAPSDSFSSSSSSSFHVDLQPYLRCSMPHKRLRVAVLLSGGVDSSVALRLLHAAGHSCTAFYLKIWFQEDFENFWSECPWEEDLKYANAVCNQVEVPLEVVHLTDEYWNNVVSYIIEEYRCGRTPNPDVLCNTRIKFGAFLDAISGMGFDYVASGHYANVIHPCADQMEEPSVLELSQDMVKDQTYFLSHLPQSQLKQLLFPLGRISKDEVRRLATEFDLPNKDRKDSQGICFLGKIRFSEFVARHIGEREGIILEAETGDFLGKHRGFWFYTIGQRQGLRLPGGPWYVVEKDIKNNVVFVSRNYFSFDKTRRLFRVSSLKWLSGLPATQISKLQCKVRHGPGFYDCSFEMEVGKDGQESVAVVRLSEDDQGLAAGQFAAFYEGRKCIGSGVILESWDDQSFPVCAKALEIARMEDKSKLGNPVKIKVKSEEVFVSTEVASKAC

>XP_009376883.1 tRNA-specific 2-thiouridylase MnmA-like [Pyrus x bretschneideri]

MAVWQLLRTVLSGGSSSSSTVTAISSSPPKPKTPISNQHSSPNLHLPKRCRLLYNVYATKPMARLIPMAMAASLLPHITSHTLPLKPYLWPFPRSPTPTTRLFVRPFSLSARSVNCFSVPQTPRSSNSFNGDVGPYLSCSMPHQRLKVAVLLSGGVDSSVALRLLHAAGHSCTAFYLKIWFQEDFENFWSECPWEEDLKYAKAVCNQVDVPLEVVHLTDEYWKNVVSYIIEEYQCGRTPNPDVLCNTRIKFGAFMDSINSMKFDYVASGHYANVVHPPASQTDKDSVLELSQDMVKDQTYFLSNLSQAQLRRLIFPLGCLSKDEVRKLATKFDLPNKDRKDSQGICFLGKIKFSEFIARHIGEKEGVILEAESGDFLGNHRGFWFYTIGQRQGLRLPGGPWYVVEKDIKNNVVFVSRNYFSVDKRRRLFRVGSLKWLNGLSPNQISQLECKVRHGPAFCKCSFIMEPGEDGHEDVAVVHLSQDDQGLAAGQFAAFYQGRTCLGSGVILESWDDQGFPVCARAHEIARMEDKSILGKPVKIKVKPENLLRESGEVDGVELCGGLKNPGIAATKQPMQVSQ

>NP_175542.2 tRNA (5-methylaminomethyl-2-thiouridylate)-methyltransferase [Arabidopsis thaliana]

MFSAVAKPFLSPSPPLFRLPLSSISISFRVFPSFFSSSSSSQLPSPQSSNPITPLISSNRNYSRHAFIVDQYLSCSMPEKPLRVAVLLSGGVDSSVALRLLHAAGHSCTAFYLKIWFQEGFENFWNQCPWEDDLKYAKHVCEQVDVPLEVVHLTDEYWERVVSYIIEEYRCGRTPNPDVLCNTRIKFGAFMDAISDMEYDYVGSGHYAKVVHPPADQNDASSVLELSQDMVKDQTYFLSHLSQTQLKRLLFPLGCVKKDEVRKLATQFDLPNKDRKDSQGICFLGKIKFSDFVCRHIGEMEGIILEAESGDFLGNHRGFWFYTIGQRQGLRLPGGPWYVVEKDTKNNVVFVSRNYYSIDKRRRIFRVGSLRWNSGKPSGKVRELRCKVRHGPGFYSCSFEMEGDGDVAVVHLDEDDQGLAAGQFAAFYEGTTCIGSGVILESWDDQCFPVCAKALQLAALEDKTKLGKPVKIMTMPTMSSVEADTGEASAEQKLVNA

>NP_011304.2 Ncs6p [Saccharomyces cerevisiae S288C]

MSFTAPSDPVNKPTKVKVSQLCELCHSRKALIRRPKNLSKLCKQCFCLVFETEIHNTIVANNLFQRGEKVAVGASGGKDSTVLAHMLKLLNDRYDYGIEIVLLSIDEGIIGYRDDSLATVKRNQQQYGLPLEIFSFKDLYDWTMDEIVSVAGIRNSCTYCGVFRRQSLDRGAAKLGISHVVTGHNADDMAETVLMNILRGDVARLEKSTAIITQSSGSPIKRSKPFKYSYQKEIVLYAHYMKLDYFSTECTYAPEAFRGTAREYMKNLEAVRPSCIIDIIQSGENLALKAKKSNAGKRVVKFVDGNRCARCGYLSSNNICKACMLLEGLEKSRAQVAIENDTSADGAALKLRALEKLSF

>NP_660275.2 cytoplasmic tRNA 2-thiolation protein 1 [Homo sapiens]

MPAPPCASCHAARAALRRPLSGQALCGACFCAAFEAEVLHTVLAGRLLPPGAVVAVGASGGKDSTVLAHVLRALAPRLGISLQLVAVDEGIGGYRDAALAAVRRQAARWELPLTVVAYEDLFGGWTMDAVARSTAGSGRSRSCCTFCGVLRRRALEEGARRVGATHIVTGHNADDMAETVLMNFLRGDAGRLARGGGLGSPGEGGALPRCRPLQFASQKEVVLYAHFRRLDYFSEECVYAPEAFRGHARDLLKRLEAARPSAVLDLVHSAERLALAPAARPPRPGACSRCGALASRALCQACALLDGLNRGRPRLAIGKGRRGLDEEATPGTPGDPARPPASKAVPTF

>NP_001015743.1 cytoplasmic tRNA 2-thiolation protein 1 [Xenopus tropicalis]

MPVNCSSCEERRAVLRRPKTGHSLCKDCFFHAFEEEIHHTIVSAKLFNPGEKVGIGASGGKDSTVLAHVLKVLNERYAYGLDLILVSVDEGISGYRDDSLETVKRNQQQYELPLKIVSYQELYGWTMDQIVKQVGLKNNCTFCGVFRRQALDRGAMMLGVNKICTGHNADDIAETVLMNFLRGDIARLRRCTSITTGSEGAIPRCKPLKYAYEKEIVLYAYFKKLDYFSTECIYSPNAYRGHARAFLKDLEAIRPSSIMDIIHSGENLSVKEDVRMPVQGTCTRCGYISSQSLCKACVLLEGLNRGLPKLGIGKHHKLHHKLLSQEPLSEQEERKLKAVDF

>NP_001099721.1 cytoplasmic tRNA 2-thiolation protein 1 [Rattus norvegicus]

MPAPTCFSCHKARAALRRPRSGQALCGPCFCAAFEAEVLRTVLAGHLLPQGAVVAVGASGGKDSTVLAHVLRELAPRLGITLHLVAVDEGIGGYRDAALEAVRSQAARWELPLTIVAYEDLFGGWTMDAVARSTAGSGRSRSCCTFCGVLRRRALEEGARLVGATHIVTGHNADDMAETVLMNFLRGDAGRLARGGVLGSTGEGCALPRCRPLQFASQKEVVLYAHFRHLRYFSEECVYAPEAFRGHARDLLKLLEAARPSAVLDLVHSAERLALAPAAKPPPPGTCSRCGALASNKLCQACALLDGLNRGLPRLAIGKGRRVLQVEPPPLGNPSRVTSDPVALPEPCTCKQSKDESNPCGNGGDRAGATCVSKSDLSPVAE

>NP_663557.1 cytoplasmic tRNA 2-thiolation protein 1 [Mus musculus]

MPAPTCFSCHKTRAALRRPRSGQALCGSCFCAAFEAEVLHTVLAGHLLPPGAVVAVGASGGKDSTVLAHVLRELAPRLGITLHLVAVDEGIGGYRDAALEAVRSQAARWELPLTIVAYEDLFGGWTMDAVARSTAGSGRSRSCCTFCGVLRRRALEEGARLVGATHIVTGHNADDMAETVLMNFLRGDAGRLARGGVLGSTGEGCALPRCRPLQFASQKEVVLYAHFRHLRYFSEECVYAPEAFRGHARDLLKLLEAARPSAVLDLVHSAERLALAPAAKPPPPGTCSRCGALASHKLCQACALLDGLNRGLPRLAIGKGRRVLQVEPPQPGNPSLVTSDPVAPAGPCTCKQPKDKANPCGNGGDRAGATCVSQCDLSPGNGEDRAGATCVSQRDLSLGNGGDRAGATCVSQCDLSPVAE

>WP_010902098.1 putative tRNA 2-thiolation protein NcsA [Halobacterium salinarum NRC-1]

MDCTKCDRDAVMHAAYSGAHLCEPHFLDSVERRVRRRIRTDNMLSPDTTPDDPETWVIGLSGGKDSAVLTDLIEETFRDDPRVELIALTIHEGIAGYRDESLDACTTLAADRDIRHELVTYREEFDVAMDDVADDDPLDMAPCAYCGVFRRDLLAKYADEYDADKLLTGHNLDDEAETALMNILEGNVTQIATHFDASLGSFDDRAELDAMIPRAKPLRDIPEKEVALYAQLRDLPVHMAECPHASESFRSDLQEVLLELEDRRPGTRHSIMAGYEELAGIAADEYNTDDADVGECSDCGAPTTRDRCRKCTLVDAVTSV

>WP_004044353.1 putative tRNA 2-thiolation protein NcsA [Haloferax volcanii DS2]

MECDKCGRDAVMHAAYSGAHLCDDHFCASVEKRVRRRIREDNMLPRDASPENPQTWVIGLSGGKDSVVLTHILDDTFGRDPRIELVALTIHEGIEGYRDKSVDACVELAEDLDIHHELVTYEDEFGVQMDDVVEKDPENMAACAYCGVFRRDLLERFADELGADKLLTGHNLDDEAQTALMNFFEGDLKQVAKHFDASIGDFEKRRDAGEFIPRAKPLRDVPEKEVALYAHLKDLPAHITECPHSSEAYRGEIQQLLLKLEENHPGTRHSIMAGYEELAELTAREYRGEGRVDLNDCERCGSKTAGDVCRKCRLIESIEAV

>QOS10697.1 putative tRNA 2-thiolation protein NcsA [Haloferax gibbonsii]

MECDKCGRDAVMHAAYSGAHLCEDHFCASVEKRVRRRIREDNMLPRDASPENPQTWVIGLSGGKDSVVLTHILDDTFGRDPRIELVALTIHEGIEGYRDKSVDACVELAEDLDIHHELVTYEDEFGVQMDDVVEKDPENMAACAYCGVFRRDLLERFADELGADKLLTGHNLDDEAQTALMNFFEGDLKQVAKHFDASIGDFEKRRDAGEFIPRAKPLRDVPEKEVALYAHLKDLPAHITECPHSSEAYRGEIQQLLLKLEENHPGTRHSIMAGYEELAELTAREYRGEGRVDLNDCERCGSKTAGDVCRKCRLIESIEAV

>Toxoplasma gondii TGGT1_309110

MFLSSLGGCKSLSAVLFLLLFVLAPRQASPLATSVASAAGSSAWLPAALPRSSGVRPPDQSRRLGRSAPRGNADNLQQLPSQNGVRRRRSVSWARGLPALFRAAADEGTEGGTRSDPREDDACSLLPSSHRPSWQLRGDAAGASLNEKSGKTRRRSGSHPWVRSVAKRRFPRWTYTSLSHHRGVPCASLLPHRCGFCPSSSFLNSARFCPVIGEDTAVRGVLAGGDGVWSHVVSGSAARCDAGRPRQEKAGVAGRKRPHTAVFFISNFAPCGASTSSRDCFANSAEDRRGAPVGPPLSPSLRSLSRSSLRSSARDPFSPATPLASSGSLPPRMRELLSELRRQKDLHAVFEKLVSFASSVPLYPARDASAVSSRASTASPPSPPRPVPSASLSPAPPPKATETSGRADDRLLPPAQLLQQPRREASEEADEEGDSPEAWERVAGCAALVRIRVCLRRVLSPKREGESRACSVFREADGRQEPQQGAVASLSVAASSSSGASCHDVEAAKKERRELRREDRDGEDERRRWELRVDLRGWSDSLVVRAWLAILVVGLNNAAPDTVLALSTDDILREAGLMPSSTPSGGKDHKDTEKETQGEPQKQTEEGEDGQREAEEEAEEEEEEERKGSKRESEEERLQEGEERVREEETRKCREEEKRRLVVPQGLEFMLRSIQRQVREQLSRLAEEEKNGGAPDGKVRKSKTDRDASRDLTGETRTRTDENGVQRSVLHRCRNDAETEADSHHVSSSLSSSLPPSQPHLSSPSSSSLSSSSSPSSSSSSSSSAAPDLFASTTETCEEKRELRRSAASSPPQVAVLLSGGVDSSVSLCLLQQRGFAPQAFFIKVWLPELLLVSRHLNRLLDSGLAPAAAGGCGWERDLLFADQVCRQARVPLEVLPLQEAYWEGVVQQMLDEARQGLTPNPDWWCNQRVKFGAFLDLLDGRETRFSARRIAGESEGENEKEEADMPFLRNSSRWTGAVASGHYARVVRAAETSRRSEEGEDTEDTDEDGEEDRGDKERGSEEERRTRLFRGKDRRKDQSYFLSGLSQRQLRRLVTPVGDMEKVEVRRLAAALDLPTARRQDSQGLCFLGNLSLSFFFRHFLGSSTGPVLHFPSCLALGSHDGLWNFTVGQRKGVTPCIDVARVRRLSSLPDSSLTPHAASVDSEGSEEQTARAGDSWRRQARAPPGKPTKASATGQGRQAADDEERRLLEAKCHRDGDDSCVRSANHTVRGRLECTDNADRPDGRVPVLSEGNEAPSSSSCSSSAEANEGQEEGGDRQSRLSSSSFAACLSGSPQNLFEPANPFSRTGLGAASLAGRWVVAAKHPPSNALFVVSEKEMKAAAAVAESVGYSLDVLAAGPGVRTLGDSQTYLLALLLTLQQKFLRVDNIQWISHPPCAACNAEEQPVSRDPSFLDTLLGSRDPRARLRGDEKSLETADEFAFLRWALEGSRDKRPRLYDVQVRHAAGTACAAIHRRVRLCLFPPERRSPSFLSPSAVSEESGGRRTESRAPFGPSRAETSAGSSGAWTAWIELAEPDEGLAPGQIAAIYEGEECLGAGRISARQGQMAVEAALRSAGLN

>Toxoplasma gondii TGGT1_309020

MDSLHASSALSSSSSSSFSASSSSSSSSSSSSSSSSSSSSSSSAFSSSSSASSSFSSASSVRFSPSRCQFCGSPRVSLLRVASRLRSCRECFVRAFEDDVSAFIRRFSLFQRGQKVAVCVSGGKDSAVLLHVLHTLNARENLGLSLHLLAVDEGIKGYRDHALAAVRRNSEVYNLPLHVVSYASLYHGWTMDRIAQQALLGKDGEPPVSRKAAEAPSSSSPSSASSGAPERPAASCCSSLSPATQSCGCSSGGGASVGPGESAEAARPGRAEKKRETQAGRSDDFTHSCTFCGIFRRQAFERGAQDIGADVLCTGHNADDGAETFLMNILRGDMQRLPVSGAPLTGSREGPSVMRVKPLLASYQREVVLYAHFNRLDYFATECTYSGAAYRGLVRNFLSSLQDNQHQQRVLDLLHAARKLWVPSRVSSEAAKDAGDSEARREGRSGDSDMAGRTQSVRRKGGGEEAAGTEVDVFLRETHENAFRECTATAATHDALQASSLSPASAYKRETEAATASSLGGDLSVPSNASSASFRLSNAGCGDRGEGGLEKNESGTGPARNGQLRPCVLCGFLTKNDLCRACALVQALNENKLDFVGMNARKGNKLMRGNERGNHEKGKASLCSERRDSIKPAVVRKENTRPSDLASQTPGLRGSLAW

>Toxoplasma gondii TGGT1_294380

MQFHRQALPPSLAASSLLPRSSLSVRLLSQIYEDTLGEAYGLETPFSSSSSLSSKPSYRPLLYADYAASGRALKSVEEFLLLRVLPLYGNTHTQESATGKQANQLLEEARHIVKMYFNCSHKDAVIFCGGGASAAVSKFLQILLLSAPHAACAWASSAVCGDTGRSRSREPVFSSSPSSSFLSTFFALFEEDRWGSFVCRACGARLKTAAHARRHMPVHPHFKFLSPVSSSSSSSSSSSSPSSPSSSSSNVPAGVCCSSRSPLAASEVPEGEALRGGEKRRARVHLRIHFFVDPCCHHSSFLPFKELATASGLASASSLQASSDSSVTGCFPEKEQTISTECAEGRYSYRGAAERPQGRQTTETAGTFRETPEPPVDMHFFFSFFDLDMRTATLAPLSVLEKLREEEQVCARLNDACRERDSTSATTAKQNHSDIDGKRRHEDIYVSLPVCIFSAASNVTGLISPTLPSSSPSSSSSPSFASSPSFAFSPFSASSLSDRSKLSMAQLNRIVHHFGGVSCWDFAGAASHIGADVNWAGGGGRLRLPVDGGRVAGGREGVETGRCELQTREHKLCVEIDEQGAGADVVFFSPHKLLGGPGSCGVLLLKKQLLLSDLPAHPGGGSVYLVSGHKAAVYTLDRSEHREEAGSPNLLAIVRAAAAIRVLQQLPMDLVRTREELWTQKLLHRLSSHPRIQIVGALHPRVGIVSLLFRYGNANHPTEPTHAHTPTDCRCLRRNYAFGGGLYLHQNFVVALLNDLFGIQVRGGCLCASPYTSFLLSIDPKLLETIEAMLLETGQDVFRPGVVRVSLHGLMRTEELDKLSAALLWVASNGWKLMPRYEVTPESGQWRVKGWAGRREEAVRTWISEASLLPQGDSEEKGDRTKNSTVFEGTIARCNETIEEMLNFANRVLSSHLSHFHASAAPSFHLLHLPPQNSSMSPPLGEKRFSAATAPLQHAPPGPAPPRGDRGDSRDSGGSRGCEGDSGEASRDSEESREQLTTLGEELLHSSSSPAWKPAASAAETAATLVWFALPADAAYSISLWSRAGGSRETDVREISTSEVSQILDAPHALSPEALPFDRELADLSPRVSASAIRWLPGVSGETRTSNADGKAEKGSDGKIPALQRAEHSCFQLRLFTGANCCSCWSHSDEARLRAVEDGQEAQGDAAEVAHAEKKQESDTVEEGDWLLVSEKTENAQTDGRTVDTPETSAAAAQVDDLRRQVAALREALEREREKSERLREKREYTGKKANSNAKRGGRAALETETHENIHKTADSTQEANACSKTSRSSFSPSSPSCSSSPPSSSPSCSSSPPSSSPSPARSFLPILQIPRTLRRTVGEAIRSFDMIRDGDRLLVGVSGGKDSLTLLHVLRDLRRRAPIHFSLAAATVDPVTPEFCPQPLIKYMEDLGIRYHYLRLPIMALAKERMQRQSICAFCSRLKRGLLYSCMRKHGYNVLVLGQHLDDACESFLMSAFHNGVLNTMKGHYVNADGDLRICRPLITTREKETAEFAARHRLPVIADNCPACFAAPKERHRMKMLLSEQEMEFPHLFQNLLKSLTPLLAISAAENRPQKAAVRRNRIPAEGSSNEARERGEKRERMPDRADEDAAGSVEPLFTTVEDFEEDSVETLLRHAGGREEGGKKMRGRDQSGESGDASELSGEKEQEGGSRAEEEDEAAELVVTACGVGSDGVCRRVKS
