# Supplementary material for: The first apicoplast tRNA thiouridylase plays a vital role in the growth of Toxoplasma gondii
Source: Front Cell Infect Microbiol. 2022 Aug 15;12:947039. doi: 10.3389/fcimb.2022.947039 (PMC9420914; doi:10.3389/fcimb.2022.947039)
Supplement: Supplementary Table S1 — Gene contents of the Toxoplasma gondii apicoplast genome. [file Table_1.docx]

Table S1 Gene contents of the *Toxoplasma gondii* apicoplast genome

| **Class** | **Gene** |
| --- | --- |
| Ribosomal RNA | *SSU*, *LSU*, *LSU*, *SSU* |
| Transfer RNA^a^ | Ile^AUC^Ala^GCA^Asn^AAC^Leu^CUA^Arg^AGA^Val^GUA^Arg^CGU^Met^AUG^Thr^ACA^  His^CAC^Cys^UGC^Leu^UUA^Met^AUG^Tyr^UAC^Ser^AGC^Asp^GAC^Lys^AAA^Glu^GAA^  Pro^CCA^Phe^UUC^Gln^CAA^Trp^UGG^Gly^GGA^Ser^UCA^Thr^ACA^Met^AUG^Arg^CGU^  Val^GUA^Arg^AGA^Leu^CUA^Asn^AAC^Ala^GCA^Ile^AUC^ |
| Ribosomal proteins | *rps*2, 3, 4, 5, 7, 11, 12, 17, 19  *rpl*2, 4, 6, 11, 14, 16, 36 |
| RNA polymerase | *rpo*B, *rpo*C1 |
| Other proteins | *Clp*, *tufA* |
| Unassigned ORFs | 5 ORFs (ORF B, C, D, E, F), ycf24 homolog |
| misc_feature | *rps*8, *rpo*C2 |

^a^ Three-letter amino acid code
