# Supplementary material for: The first apicoplast tRNA thiouridylase plays a vital role in the growth of Toxoplasma gondii
Source: Front Cell Infect Microbiol. 2022 Aug 15;12:947039. doi: 10.3389/fcimb.2022.947039 (PMC9420914; doi:10.3389/fcimb.2022.947039)
Supplement: Supplementary Table S2 — Sequences for primers used in this study. [file Table_2.docx]

Table S2 Sequences for primers used in this study

| Name | Sequences |
| --- | --- |
| 309020-SgRNA-F | AGTTCCTCGTACGTTTCATTATGG |
| 309020-SgRNA-R | AAACCCATAATGAAACGTACGAGG |
| 309110-SgRNA-F | AGTTGCTACTTCTGTTGAAGAGTTC |
| 309110-SgRNA-R | AAACGAACTCTTCAACAGAAGTAGC |
| 294380-SgRNA-F | AGTTGGATGGACGCACACGGACCG |
| 294380-SgRNA-R | AAACCGGTCCGTGTGCGTCCATCC |
| 309020-6HA-donar-F | ACACAAGACCCTCAGACCTAGCCTCGCAAACGCCTGGTTTGCTAGCAAGGGCTCGGGCT |
| 309020-6HA-donar-R | TCGCGAGGACACTTGAGTGTGCCTCATTGCGTGCGTACAAATAGGGCGAATTGGAGCTC |
| 309110-3HA-donar-F | ACAAGGGCAAATGGCCGTTGAGGCTGCTCTGCGGTCTGCGGGTCTGAACGGCGGAGAAATCTCCCTGAGA |
| 309110-3HA-donar-R | GCATGTCTCTGAATCGCTGTAGAAACGTCTGTCTGCAGGAGAGTTGTTATAGGGCGAATTGGAGCTCC |
| 294380-6HA-donar-F | GAGAGACTCTTCTGCAAAAATTGCTGAACTCTTAAAGGCAGCTAGCAAGGGCTCGGGCT |
| 294380-6HA-donar-R | TGCCTTTAAGAGTTCAGCAATTTTTGCAGAAGAGTCTCTCATAGGGCGAATTGGAGCTC |
| 309020-6HA-F | GGCTTCCTCACGAAGAACGA |
| 309020-6HA-R | AGGATACGCATAATCGGGCAC |
| 309110-3HA-F | CAGCAGAAATTCTTGCGCGT |
| 309110-3HA-R | TACATATGCCCGGGTTAGGC |
| 294380-6HA-F | CCGAGGGAAGTTCCAACGAA |
| 294380-6HA-R | ACGCATAATCGGGCACATCA |
| *TgMnmA*-SgRNA (KO)-F | AGTTGTTCGACGGCGGATGCTTCG |
| *TgMnmA*-SgRNA (KO)-R | AAACCGAAGCATCCGCCGTCGAAC |
| *TgMnmA*-KO-donar-F | GGCTCTCCGGCGAATCACGAGGAAAACGCGTCAGTCCTCGCGTCGCCGGGGGGCAAGAATTGTGTTAACC |
| *TgMnmA*-KO-donar-R | GCTCGACTTTTCCTCCTTGTCACGAGAAGATGCCGAGTCCCTCGCTCTTTACATCCGTTGCCTTTTC |
| *TgMnmA*-KO-CDS-F | ATCCTCCCCTAGAAAAACCGT |
| *TgMnmA*-KO-CDS-R | GTACGCAGCGGGAGTCATATT |
| *TgMnmA*-KO-5’F | ACGCTCGACGAATCTAGCTG |
| *TgMnmA*-KO-5’R | CCGGTGGTGCAGATGAACTT |
| *TgMnmA*-KO-qCPR-F | ACGAGGCCCGACAAGGC |
| *TgMnmA*-KO-qCPR-R | TTTTCCCCCTCGCTTTCTCC |
| DHFR-SgRNA (KO)-F | AGTTACACGCTTTGTGGTGAGAAG |
| DHFR-SgRNA (KO)-R | AAACCTTCTCACCACAAAGCGTGT |
| *TgMnmA*-COMP-donar-F | GGCTCTCCGGCGAATCACGAGGAAAACGCGTCAGTCCTCGCGTCGCCGATGGCTGGAGTTCCCGAACCT |
| *TgMnmA*-COMP-donar-R | GCTCGACTTTTCCTCCTTGTCACGAGAAGATGCCGAGTCCCTCGCTCTATAGGGCGAATTGGAGCTCC |
| *TgMnmA*-COMP-CDS-F | GAAGGCGTCGTGCAGCAGAT |
| *TgMnmA*-COMP-CDS-R | AGGCGGCGGACCTCGAC |
| *TgMnmA*-COMP-5’F | TTGCTTAGTACCTCCGACGC |
| *TgMnmA*-COMP-5’R | TGGTGAGAGAGCGAGGTGTA |
| *rps*-3-F | TCTTTACGTAAGATTTTAAAACAA |
| *rps*-3-R | ACTCCATTAATTTTACCTGAAAC |
| *rps*-7-F | TCTGGTAAAAAAAGAAAAACTGGA |
| *rps*-7-R | AATTGGTTGTGGTACTTGATAAAT |
| *rps*-8-F | ATTTAATTTCTACAACTAAAGGATT |
| *rps*-8-R | TTTATAAGCTTCTAGACCTGACAT |
| *rps*-11-F | ATATTGTTATGAAAGGAATAGGAT |
| *rps*-11-R | ACTTTGCGTTAAATCTGTTATAGA |
| *rps*-12-F | ACACGAACTCCTAAAAAACCTAACT |
| *rps*-12-R | TGACCTTCTCCAGGAATATAAGCTA |
| *rps*-17-F | ATGGTTATTAAAATAGGTTACG |
| *rps*-17-R | AATTCTTTTCTAGAATCATGAA |
| *rps*-19-F | AATTAATGGAAGTTTATACTGGTAGAT |
| *rps*-19-R | CTTCCTATTTTATCTTTTGTA |
| *rpl*-2-F | TCAGAGGAGCTGCAATGAATG |
| *rpl*-2-R | AACTGAAGCCTTTCCTTCTCCA |
| *rpl*-4-F | ACAAAAAGGATCAGGAAAAGCGA |
| *rpl*-4-R | ACCAAAAGCAACTGAACCTCCT |
| *rpl*-6-F | TTTAATGCTTAATTATAGCCATAT |
| *rpl*-6-R | AATACTATTAGCAGCTTCAATCTG |
| *rpl*-11-F | TCTTTAAGTTCTATTTTAGGACCTA |
| *rpl*-11-R | AAATTTAATACATAAGATTTATCGT |
| *rpl*-14-F | TATGTTTCAGATAATACTGGAGTTAAA |
| *rpl*-14-R | ACTACAAAACCATAAACTATTTCAGAA |
| *rpo*B-F | TGCGAGATGGAATACCTAAGGC |
| *rpo*B-R | AGCAGAAAACGCTGATTTACCT |
| *rpo*C1-F | ACCCATTCTCCAACGTCTACTT |
| *rpo*C1-R | TTGGGCCTTTGCTTTCTTGA |
| *rpo*C2-F | ACGACGTGTTAAATACCCAGCA |
| *rpo*C2-R | TCATGTTATGGAGCAAGAAAAGGAA |
| *Clp*-F | TGGACAACCTACAGCAATTTCTAA |
| *Clp*-R | TTCCGCTTGGACCGCATAAT |
| *tufA*-F | TGATTACTGGAGCCGCACAA |
| *tufA*-R | TGGCATTGGCCCATCTACAG |
| *SUFB*-F | ACCACCTTCTCCTAATTGATTACCT |
| *SUFB*-R | TGAAGGTTGTTCAGCTCCTATGTT |
| ORF-B-F | AAAGTTATTATAAATAAATTAAAAAAA |
| ORF-B-R | AAATTTTATTATTTCAATTAGATAGTTG |
| ORF-E-F | ATTATTTTATTTTTTATTTTTTTTC |
| ORF-E-R | TAATAGGTTATTTTTAAATTTTTTT |
| ORF-F-F | AAAAAAATATTTTGTAAAAAAATCAGGA |
| ORF-F-R | TTTATTGCTTTATAAGATAAAAAGCCAA |
